# Housing conditions and risk of incident COPD: a Danish cohort study, 2000–2018

**DOI:** 10.1186/s12889-024-19131-3

**Published:** 2024-06-27

**Authors:** Stine Kloster, Anne Marie Kirkegaard, Michael Davidsen, Anne Illemann Christensen, Niss Skov Nielsen, Lars Gunnarsen, Jørgen Vestbo, Annette Kjær Ersbøll

**Affiliations:** 1 National Institute of Public Health, University of Southern Denmark, Studiestraede 6, 1455 Copenhagen K, Denmark; 2 Department of the Built Environment, Aalborg University, A.C. Meyers Vaenge 15, 2450 Copenhagen, SV Denmark; 3 Division of Infection, Immunity and Respiratory Medicine, University of Manchester, Manchester, M13 9 PL UK

**Keywords:** Indoor environment, Built Environment, Housing, Chronic Obstructive Pulmonary Disease, Epidemiology

## Abstract

**Background:**

More knowledge is needed on the risk of developing chronic obstructive pulmonary disease (COPD) associated with housing conditions and indoor environment based on cohort studies with a long follow-up time.

**Objective:**

To examine the association between housing conditions and indoor environment and the risk of developing COPD.

**Methods:**

In this cohort study, we followed 11,590 individuals aged ≥ 30 years free of COPD at baseline. Information on incident COPD and housing conditions and indoor environment was obtained from the Danish national registers and the Danish Health and Morbidity Survey year 2000. Poisson regression of incidence rates (IRs) were used to estimate incidence rate ratios (IRRs) of COPD.

**Results:**

The overall IR of COPD was 8.6 per 1,000 person-years. Individuals living outside the biggest cities vs. living in the biggest cities (≥ 50,000) had a lower risk of COPD (200-4,999; IRR 0.77 (95% CI 0.65-0.90). Individuals living in semi-detached houses had a higher risk compared to individuals living in detached houses (IRR 1.29 (95% CI 1.07-1.55)). Likewise, individuals living in rented homes had a higher risk (IRR 1.47 (95% CI 1.27-1.70)) compared to individuals living in owned homes. The IR of COPD was 17% higher among individuals living in dwellings build > 1982 compared with individuals living in older dwellings (< 1962), not statistically significant though (IRR 0.83 (95% CI 0.68-1.03)). Likewise, the IR of COPD was 15% higher among individuals living in the densest households compared with individuals living in the least dense households, not statistically significant though (IRR 1.15 (95% CI 0.92-1.45)). This was primary seen among smokers. There was no difference in risk among individuals with different perceived indoor environments. Overall, similar patterns were seen when stratified by smoking status with exception of perceived indoor environment, where opposite patterns were seen for smokers and never smokers.

**Conclusion:**

Individuals living in semi-detached houses or rented homes had a higher risk of developing COPD compared to individuals living in detached or owned homes. Individuals living in cities with < 50.000 residents had a lower risk of COPD compared to individuals living in cities with ≥ 50.000 residents.

**Supplementary Information:**

The online version contains supplementary material available at 10.1186/s12889-024-19131-3.

## Background

Chronic obstructive pulmonary disease (COPD) ranks as the third leading cause of mortality worldwide ([GBD 2019 Diseases and Injuries Collaborators, [1]). The age-adjusted prevalence is 3.0% in Western Europe and 4.3% in Denmark [2].

COPD results from an interplay between genetic susceptibility and exposure to environmental irritants [3]. Smoking is the most well-studied risk factor for the development of COPD. However, the importance of other environmental exposures has gained more attention lately [4–6]. Recently, a systematic review and meta-analysis associated long-term exposure to ambient air pollution to the incidence of COPD [4].

Despite most people in the Western world spend most of their time indoor, knowledge about the role of indoor environment in relation to COPD is sparse. A body of evidence indicates that housing condition and the indoor environment are associated with exacerbations of COPD [3, 7–9]. Also, older dwellings [10], home ownership [11] and mold [12] have been associated with respiratory symptoms in general. Nevertheless, less is known about how housing conditions and indoor environment are associated with the risk of developing COPD. Previously, type of housing, especially living in a mobile home, has been associated with COPD in a cross-sectional study [13]. However, the temporal relation between housing condition and COPD is of importance since individuals with existing COPD might move to a residence that better suits the limitations, they might experience due to COPD (e.g., a home without stairs) or move due to consequences of their COPD (e.g., economic). Furthermore, the sparse literature on the association between housing condition and COPD is based on self-reported information about doctor-diagnosed COPD which is prone to recall bias and diagnostic uncertainty.

Therefore, more knowledge is needed on the risk of developing COPD based on cohort studies with a long follow-up time. Thus, the aim of our study was to examine the association between housing conditions and indoor environment and the risk of developing COPD.

## Method

### Study design

The study was a cohort study combining data at the individual level from the national representative Health and Morbidity Survey year 2000 (DHMS 2000), the Danish Civil Registration System, the Building and Housing Register, the Danish National Patient Register, and the Danish Prescription Register.

### Setting and study population

A total of 16,688 individuals (≥ 16 years) participated in the DHMS 2000, corresponding to a response rate of 74.2%. Participants completed an interview with questions about; e.g., demography, health, and indoor environment. Data were collected in three rounds during February, May, and September in 2000 by trained interviewers in the home of the respondent.

Details of the questionnaire design and sampling are described elsewhere [14, 15]. Individual-level linkage of participants from the DHMS 2000 and Danish national health and administrative registers was enabled by the unique and permanent identification number assigned to all Danish residents at birth or immigration [16]. In Denmark, all people have free and equal access to health care.

The study population consisted of all individuals ≥ 30 years old and free of COPD 10 years before date of interview. COPD was defined based on register-based information see section ´Chronic Obstructive Pulmonary Disease´ and Supplementary Table S1.

### Assessment of housing conditions and indoor environment

Information about construction period, urbanization, and resident density was obtained by linkage to the Building and Housing Register [17] and the Danish Civil Registration System [16, 18] whereas information about type of housing and self-reported indoor environment was obtained from the questionnaire.

Period of construction was based on information about the year of construction and categorized into < 1962, 1962–1982, and ≥ 1983. The categorization reflected changes in the Danish regulations related to building construction.

Living in urban and rural areas was based on information from the Building and Housing Register [17] and the Danish Civil Registration System [16, 18] and categorized into rural (< 200), 200–4,999, 5,000–49,999 and ≥ 50,000 residents. For descriptive purpose only, the variable was dichotomized into ≥ 50,000 residents or not.

Home ownership was obtained from the Building and Housing Register [17] based on information about whether the home was occupied by the owner or by a tenant. Individuals in cooperative dwellings were grouped as tenants.

Resident density was based on dwelling size and number of individuals in the household. The latter was assessed yearly. Resident density was categorized into quartiles. For descriptive purpose only, the variable was split at the median value (below 55 m^2^ per person; yes/no).

Type of housing was reported in the questionnaire by the interviewer. Type of housing was categorized into detached houses, semi-detached and terrace houses, apartments, farms, and others. The category `other´ contained institutions, college, etc. For the descriptive analyses, the type of housing was categorized into apartments and other types of housing.

Perceived indoor environment was based on 13 items: Self-reported perceived annoyances within the past 14 days (12 items), and placement of the dwelling next to a trafficked road (no/yes) (1 item). Based on these items, three latent classes were identified using Latent Class Analysis. The three classes were characterized by 1) very few annoyances (88.8%, *n* = 14,829 based on most likely class membership), 2) moderate annoyances (5.9%, *n* = 980) and 3) many annoyances (5.3%, *n* = 879) (described in [19]).

### Chronic obstructive pulmonary disease

Information about COPD was retrieved from the Danish National Patient Register [20] and the Danish Prescription Register [21] from 10 years prior enrollment/baseline and until the end of follow-up (prescriptions from 1995). An incident COPD was defined as a first-time primary diagnosis of J44 (COPD), or J96 (respiratory failure) as a primary diagnosis combined with J44 as a secondary diagnosis, or J13-18 (pneumonia) as the primary diagnosis combined with J44 or J96 as one of the secondary diagnoses and/or a redemption of two prescriptions with Anatomical Therapeutic Chemical (ATC) code R03A or R03B or indication code 464 or 379 within a 12-month period [22, 23] (see Supplementary Table S1).

### Covariates

Age, sex, and socioeconomic position were identified as confounders a priori using a directed acyclic graph (DAG) [24, 25] (see Supplementary Figures S1a and S1b). When examining the association between type of housing, home ownership, resident density, or perceived indoor environment and risk of COPD, cohabitation was identified as a confounder too. For the association between perceived indoor environment and COPD, timed lived in the residence was also identified as a confounder. Depending on the exposure examined, some of the housing conditions were also identified as confounders, e.g., year of construction and urbanization were identified as confounders for the association between type of housing and risk of COPD (see Supplementary Figures S1a-S1f). Smoking was only identified as a confounder in the association between perceived indoor environment and the risk of COPD. However, since smoking is a very strong risk factor of developing COPD [26, 27] all analyses were adjusted for smoking status with exception of the association between home ownership and COPD, where smoking was identified as a mediator and therefore not controlled for. Home ownership is a known marker of socioeconomic position [28], and as smoking is more common in groups with lower socioeconomic positions, smoking was considered a mediator. Additionally, all analyses were adjusted for the calendar years (2000–2003, 2004–2007, 2008–2011, 2012–2015, and 2016–2018) since the IR of COPD varied during the study period. All confounders were assessed at baseline except for age, resident density, cohabitation, and household income which were assessed yearly and included in the analysis as time-varying covariates.

Information about age (30–39, 40–49, 50–59, 60–69, 70–79, and ≥ 80 years), sex (male and female), and cohabitation (living alone and cohabiting) were obtained from the Danish Civil Registration System [18]. Socioeconomic position was assessed by educational level and equalized disposable household income. Highest attained educational level was obtained from the Danish Education Register [29] and categorized as; elementary (International Standard Classification of Education (ISCED) 1–2), short (ISCED 3) and medium/long (ISCED 5–8). Information about equivalized disposable household income was obtained from the Income Statistic Register [30] and grouped into quintiles year by year. For some of the stratified analyses, incomes in quartiles were used. Time lived in residence prior participation was calculated based on information on moving history from Statistics Denmark and categorized as: < 3 years, 3–10 years, 11–20 year, and ≥ 21 years.

Information about smoking status was obtained from the interview by asking “Do you smoke?” and “Have you been smoking previously?”. Those questions were combined into a single variable indicating the smoking status of the respondent: 1) never smoker; 2) former smoker; 3) current smoker.

For descriptive purpose, information from the questionnaire on Body Mass Index (BMI) and secondhand smoke was included.

### Statistical methods

For the descriptive analysis, median and interquartile range (IQR) were used for continuous variables and counts with proportions were used for categorical variables. Incidence rates (IR) were used to describe the rate of COPD.

The association between housing conditions, indoor environment, and COPD was examined using a Poisson regression with COPD as outcome and logarithmic transformation of follow-up time as the offset. We adjusted the analyses for the potential confounders identified a priori (see section ´covariates´ for more information). Follow-up time was split by calendar year. Results are presented as incidence rate ratios with corresponding 95% confidence intervals (IRR and 95% CIs). All participants free of COPD were followed from the date of interview and until the occurrence of COPD, death, emigration, change of address, or end of the study (December 31, 2018), whichever came first. Analyses were weighted for non-responses by weights estimated by Statistics Denmark based on information such as sex, age, education, and income [31]. This implies that a weighting value was calculated for each respondent in the survey, and that this value indicates how much the respondent´s answer will count in the Poisson regression.

To rule out the potential influence of smoking, all analyses were further stratified by smoking status (non-smokers, former smokers, and current smokers).

All analyses were performed using STATA software (Stata Statistical Software: Release 17.0. College Station, TX: StataCorp LLC).

### Sensitivity analyses

According to the DAG, the association between resident density and COPD as well as the association between perceived indoor environment and COPD should have been adjusted for type of housing and home ownership. However, as the overlap between these two variables was high, both variables could not be included simultaneously. Therefore, the variable expected to control for the most important information was included in the main analysis (e.g., type of housing in the association between resident density and COPD). Analyses were repeated with adjustment for the opposite variable.

### Missing data

The number of missing data on covariates was low and ranged from 0 (e.g., age and sex) to 26 (e.g., smoking status) and all analyses was conducted as complete cases. Information about household income was not available for the last year of follow-up for 1,582 individuals. Since the income register is compiled at the end of year, this can occur if, e.g., individuals emigrate or die before the income register is compiled. For those individuals, the information from the previous year was used. The same applied to the number of persons living in the residence and cohabitation, where the number of missing was 1,581.

## Results

**Fig. 1:**
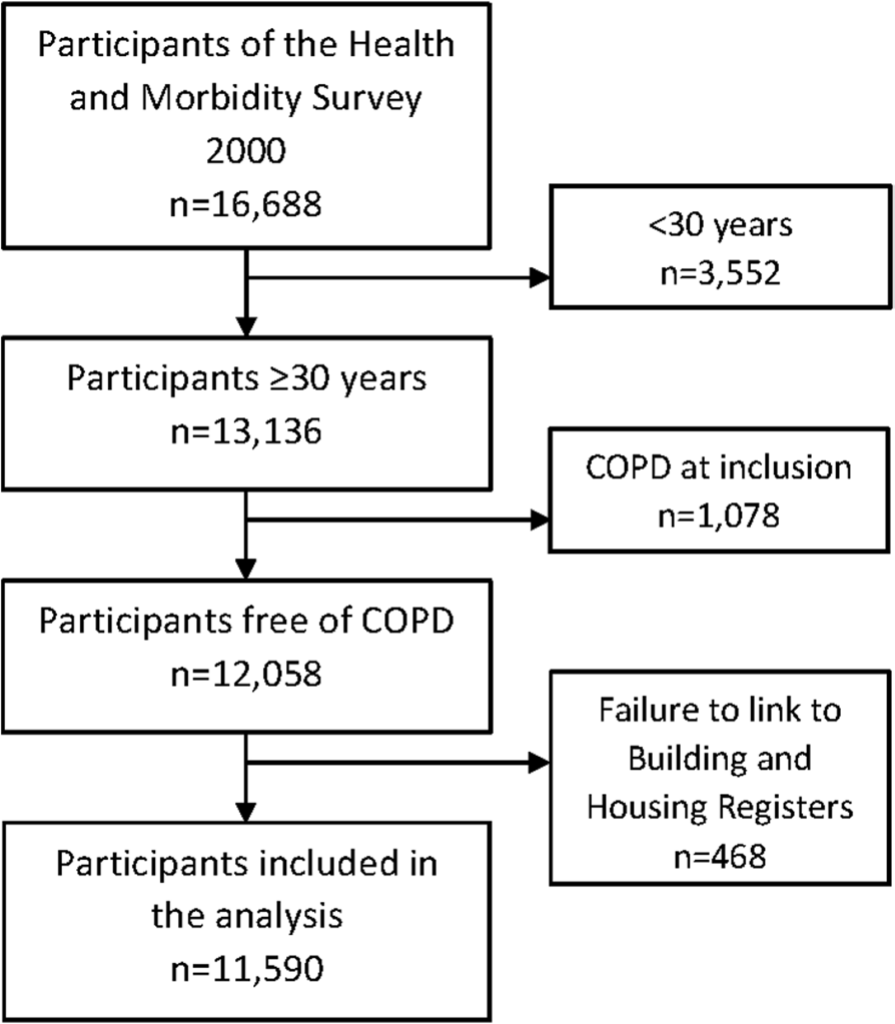
Flow diagram of individuals in the study

We included 11,590 individuals (49.4% men) free of COPD at the date of interview in the year 2000. A detailed description of the flow of individuals is given in Fig. 1. The age ranged from 30 to 98 with a median age of 51.4 years (IQR 40.7–62.8). Most individuals lived in a detached house (56.0%) and 16.8% lived in an apartment. Moreover, most individuals lived in owned homes (71.5%). In total, 23.8% of the individuals lived in cities with 50,000 residents or more, while 17.6% lived in rural areas (< 200 residents). Individuals had lived in the residence for a median time of 10.1 years (IQR 3.8–21.6) at inclusion.

Individuals living in residences built before 1962 more often lived alone (29.9%) compared to individuals living in residences built after 1962 (24.0%) (Table 1). Likewise, among individuals living in larger cities (≥ 50.000 residents), more individuals were living alone (36.4%) compared to individuals living in smaller cities (24.0%). Moreover, individuals in the larger cities had a higher educational level (29.4 vs. 21.1%) and less had lived in the same residence for a long period (≥ 21 years) (23.6 vs. 26.9%). Individuals living in apartments were in general younger (28.6% < 40 years) compared to individuals not living in apartments (22.1%). Likewise, a higher proportion were living alone (56.5 vs. 20.9%), were in the lowest income quintile (33.6 vs. 17.3%) and were current or previous smokers. Individuals living in owned homes were in general younger, with exception of the youngest (the proportion of individuals < 40 years was lower), more often cohabitating, more individuals were in the highest income quintile (25.0 vs. 7.6%), had a lower proportion of smokers (35.0 vs. 44.5%) and had lived in the residence for a longer period. Individuals living in more dense residences (< 52.5 m^2^ per person) were younger, more often cohabitating and had lived in the residence for a shorter period at baseline (25.2 vs. 16.0%; < 3 years). Individuals reporting many annoyances (Latent class number three) differed on several characteristics, e.g., more were women (57.3%), they were younger (40.3%), a higher proportion were in the lowest income quintile (27.7 vs. 19.8%), and a larger proportion had only lived in the residence for ≤ 3 years compared to individuals reporting less annoyances (Latent class one and two) (see Table 1 for more details).

**Table 1:** Baseline characteristics of the study population (*n* = 11,590)

	**All**		**Construction period** < 1962		**Urbanization** ≥ 50,000 residents		**Type of housing** Apartments		**Home owner**		**Resident density** < 52.5 m^2^/person (median)		**Perceived indoor** **environment (annoyances)**		
			yes	no	yes	no	yes	no	yes	no	yes	no	Very few	Moderate	Many
	N	%	%	%	%	%	%	%	%	%	%	%	%	%	%
**Sex**															
Men	5,728	49.4	50.6	48.2	48.6	49.7	45.3	50.1	51.2	44.9	50.8	48.0	49.6	50.8	42.7
Women	5,862	50.6	49.4	51.8	51.4	50.3	54.7	49.9	48.8	55.1	49.2	52.0	50.4	49.2	57.3
**Age (years)**															
30–39	2,696	23.3	25.8	21.0	27.2	22.0	28.6	22.1	22.5	25.0	36.8	9.6	22.3	26.8	40.3
40–49	2,716	23.4	23.4	23.5	21.4	24.1	16.4	24.9	25.4	18.5	31.0	15.8	23.3	26.0	22.4
50–59	2,714	23.4	21.2	25.5	21.1	24.1	18.6	24.4	26.0	17.1	16.1	30.8	23.8	22.2	15.4
60–69	1,659	14.3	13.2	15.4	12.7	14.8	13.8	14.4	14.6	13.8	7.9	20.8	14.5	13.1	11.9
70–79	1,151	9.9	10.8	9.2	11.4	9.5	14.3	9.0	8.0	14.8	5.6	14.3	10.2	7.7	6.8
≥ 80	654	5.6	5.6	5.5	6.2	5.5	8.4	5.1	3.4	10.7	2.5	8.6	5.8	4.2	3.3
**Cohabitation**															
Cohabitating	8,465	73.0	70.1	76.0	63.6	76.0	43.5	79.1	83.7	47.0	86.8	59.5	73.7	68.6	63.9
Living alone	3,125	27.0	29.9	24.0	36.4	24.0	56.5	20.9	16.3	53.0	13.2	40.5	26.3	31.4	36.1
**Educational level**															
Elementary	4,080	35.2	36.1	34.4	28.1	37.4	37.0	34.9	32.2	43.0	31.3	39.2	35.4	31.8	35.4
Short	4,834	41.7	39.9	43.5	42.6	41.5	39.7	42.1	43.3	38.0	45.1	38.4	41.6	46.1	38.0
Medium/long	2,668	23.1	24.0	22.1	29.4	21.1	23.3	23.0	24.6	19.0	23.6	22.4	23.0	22.1	26.6
**Household income**															
Lowest quintile	2,326	20.0	23.1	17.1	19.7	20.2	33.6	17.3	12.8	37.6	19.0	20.9	19.8	19.0	27.7
Second quintile	2,313	20.0	21.8	18.2	19.4	20.1	23.9	19.2	17.9	25.3	23.1	16.8	19.8	20.5	22.8
Third quintile	2,313	20.0	20.1	19.9	18.9	20.3	17.8	20.4	21.1	17.4	23.9	16.0	19.7	21.4	22.8
Fourth quintile	2,322	20.0	18.6	21.4	20.5	19.9	14.0	21.3	23.3	12.2	20.7	19.4	20.2	21.6	14.7
Highest quintile	2,316	20.0	16.4	23.4	21.5	19.5	10.7	21.9	25.0	7.6	13.3	26.8	20.5	17.6	11.9
**Body Mass Index (kg/m**^**2**^**)**															
< 18.5	254	2.2	2.2	2.2	2.6	2.1	2.9	2.1	1.9	3.0	2.2	2.3	2.2	2.2	4.2
18.5–24.9	5,760	50.6	51.1	50.2	55.0	49.2	51.6	50.4	51.1	49.5	51.8	49.5	50.4	53.5	51.7
25–29.9	4,163	36.6	36.1	37.1	33.3	37.6	33.4	37.2	37.1	35.3	35.1	38.1	36.8	33.3	35.6
≥ 30	1,201	10.6	10.6	10.5	9.1	11.0	12.1	10.3	9.9	12.2	11.0	10.1	10.6	11.0	8.5
**Smoking**															
Never	4,155	35.9	34.9	37.0	33.7	36.6	30.7	37.0	38.0	31.0	35.0	36.9	36.2	34.8	29.8
Former	3,043	26.3	25.2	27.4	27.5	25.9	23.6	26.9	27.0	24.5	25.3	27.4	26.5	26.8	22.1
Current	4,366	37.8	40.0	35.6	38.8	37.4	45.7	36.2	35.0	44.5	39.7	35.7	37.3	38.4	48.0
**Time lived in residence (years)**															
< 3	2,403	20.7	20.8	20.4	22.1	20.3	29.3	19.0	15.8	32.3	25.2	16.0	20.0	25.9	31.9
3–10	3,701	31.9	32.0	31.9	34.9	31.0	38.0	30.7	29.7	37.5	37.7	26.1	31.6	32.8	38.9
11–20	2,458	21.2	20.2	22.2	19.4	21.8	16.2	22.3	23.4	16.0	22.6	19.9	21.6	17.7	16.3
21	3,028	26.1	27.0	25.5	23.6	26.9	16.5	28.1	31.1	14.2	14.4	38.0	26.8	23.6	12.8

Current smoking (37.8%), previous smoking (26.3%), and secondhand smoking (40.1%) were common at baseline. Overall, 47.2% of the individuals had a BMI ≥ 25 kg/m^2^. The distribution of covariates for each group of the examined exposures are displayed in supplementary (Tables S2a-S2e).

Median follow-up time was 9.9 years (IQR 3.5–18.3). During the study period from the year 2000 to 2018, a total of 1,033 Individuals were diagnosed with COPD (8.9%), corresponding to an overall IR of COPD at 8.6 per 1,000 person-years.

### Association between year of construction and COPD

**Fig. 2:**
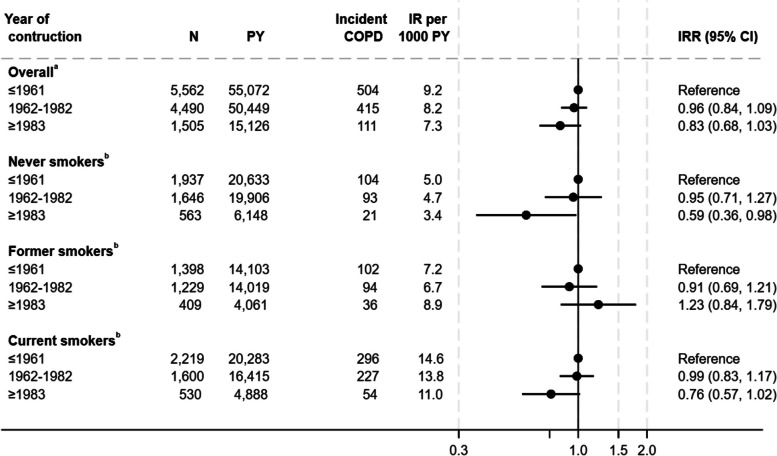
Association between construction year and incidence rate of Chronic Obstructive Pulmonary Disease ^a^Adjusted for educational level, household income, age, sex, smoking status, and calendar year. Weighted for non-response. ^b^Adjusted for educational level, household income, age, sex, and calendar year. Weighted for non-response COPD Chronic Obstructive Pulmonary Disease, CI confidence interval, IR Incidence rate, IRR Incidence rate ratio, PY Person years

The IR of COPD was 17% lower among individuals living in dwellings built after 1982 compared with individuals living in dwellings built before 1962, (Fig. 2), only borderline significant although (IRR 0.83 (95% CI 0.68–1.03)). When stratified by smoking status, the same pattern was seen among never smokers and current smokers.

### Association between urbanization and COPD

**Fig. 3:**
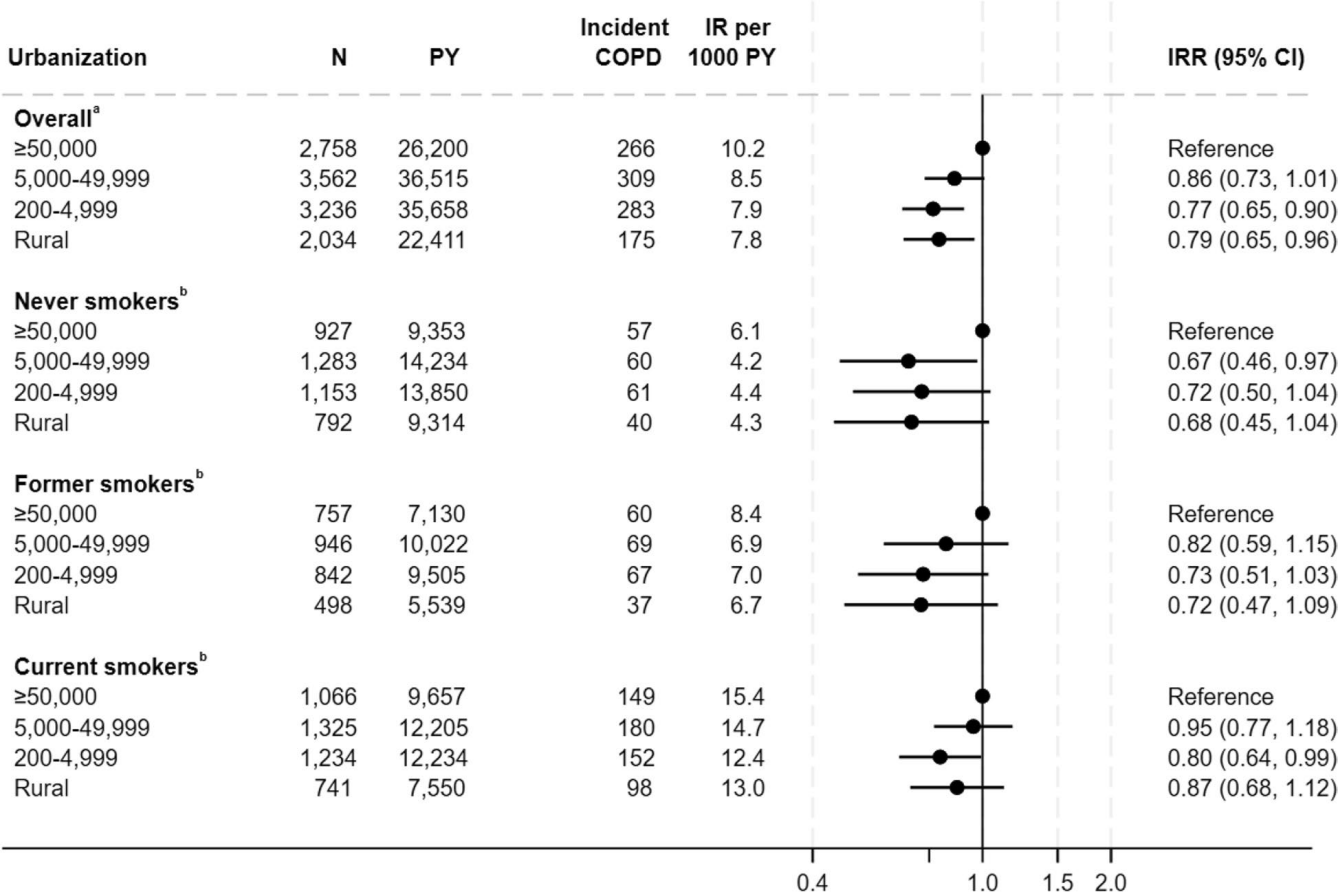
Association between urbanization and incidence rate of Chronic Obstructive Pulmonary Disease ^a^Adjusted for year of construction, educational level, household income, age, sex, smoking status, and calendar year. Weighted for non-response. ^b^Adjusted for year of construction, educational level, household income, age, sex, and calendar year. Weighted for non-response COPD Chronic Obstructive Pulmonary Disease, CI confidence interval, IR Incidence rate, IRR Incidence rate ratio, PY Person years

The IRs of COPD were lower among individuals living outside the biggest cities compared to individuals living in cities with ≥ 50.000 residents; the IRs were 14%, 23%, and 21% for individuals living in cities with 5,000–49,999 citizens, 200–4,999 citizens and in rural area, respectively (Fig. 3). However, insignificant among individuals in cities with 5,000–49,999 citizens. When stratified by smoking status, the same overall pattern was seen.

### Association between type of housing and COPD

**Fig. 4:**
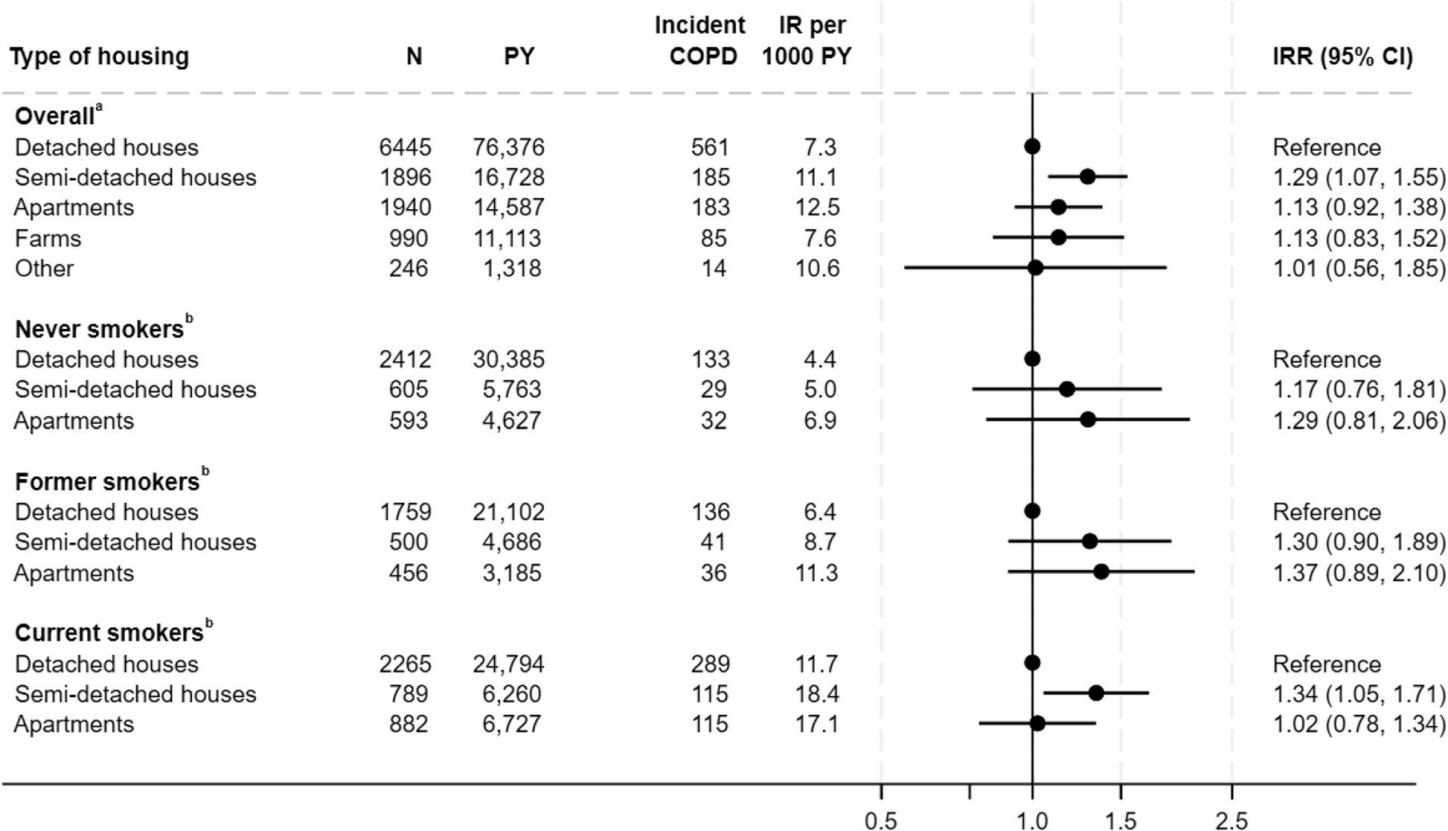
Association between type of housing and incidence rate of Chronic Obstructive Pulmonary Disease ^a^Adjusted for year of construction, urbanization, educational level, household income, age, sex, cohabitation, smoking status, and calendar year. Weighted for non-response. ^b^Adjusted for year of construction, urbanization, educational level, household income, age, sex, cohabitation, and calendar year. Weighted for non-response COPD Chronic Obstructive Pulmonary Disease, CI confidence interval, IR Incidence rate, IRR Incidence rate ratio, PY Person years

Individuals living in semi-detached houses had a higher risk of COPD (IRR 1.29, 95% CI 1.08–1.55) compared to individuals living in detached houses. The IR was 13% higher among individuals living in apartments compared with individuals living in detached houses, insignificant though. (IRR 1.13, 95% CI 0.92–1.38) (Fig. 4). When stratified by smoking status, similar patterns were seen, with exception of current smokers living in apartments who seems not to have the same IR of COPD as individuals living in detached houses (IRR 1.02, 95% CI 0.78–1.34).

### Association between home ownership and COPD

**Fig. 5:**
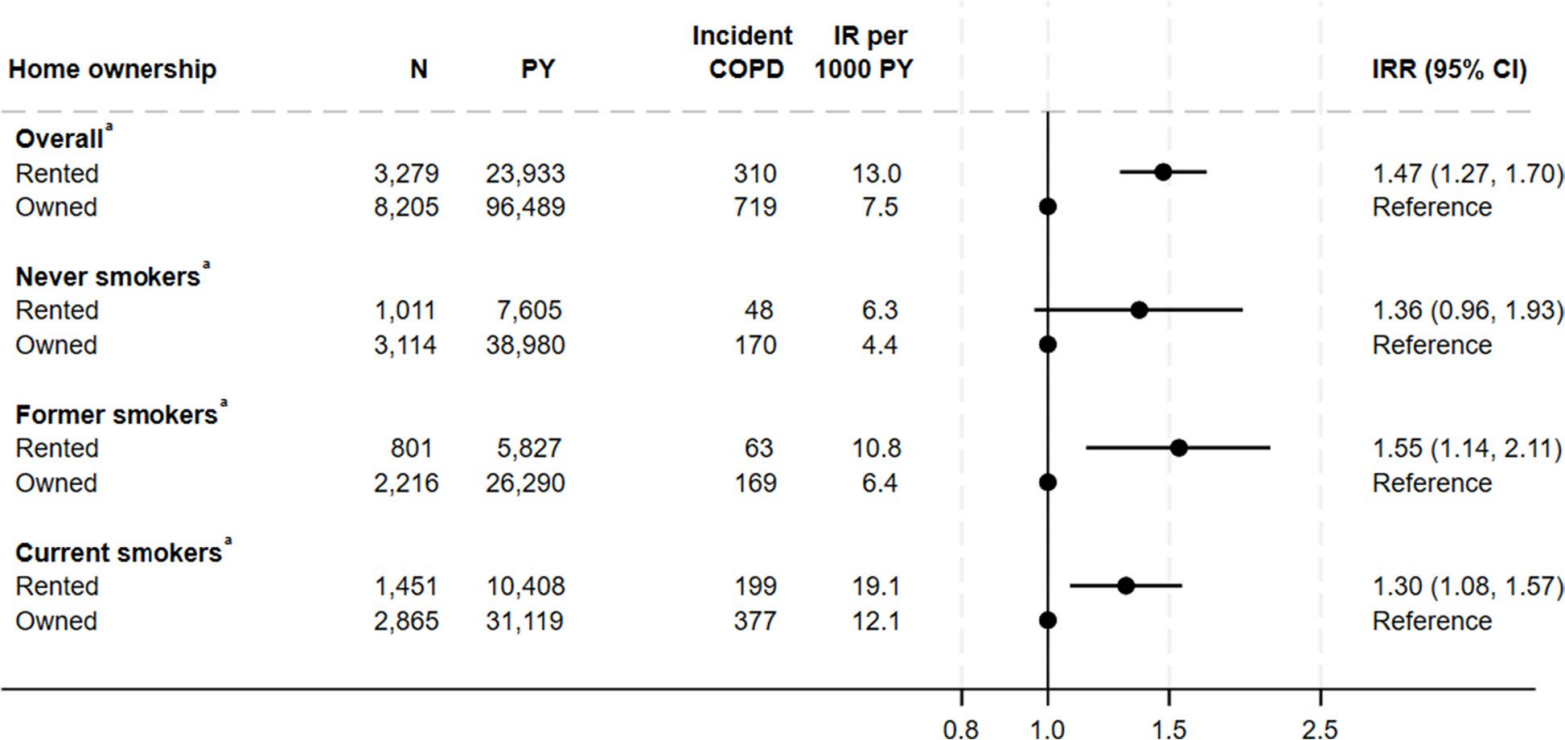
Association between home ownership and incidence rate of Chronic Obstructive Pulmonary Disease ^a^Adjusted for educational level, household income, age, sex, cohabitation, and calendar year. Weighted for non-response COPD Chronic Obstructive Pulmonary Disease, CI confidence interval, IR Incidence rate, IRR Incidence rate ratio, PY Person years

Individuals living in rented homes had a higher risk of COPD with an IRR of 1.47 (95% CI 1.27–1.70) compared to individuals living in owned homes (Fig. 5). When stratified by smoking status, similar results were seen. However, the IR was not higher among individuals living in apartments among smokers.

### Association between resident density and COPD

**Fig. 6:**
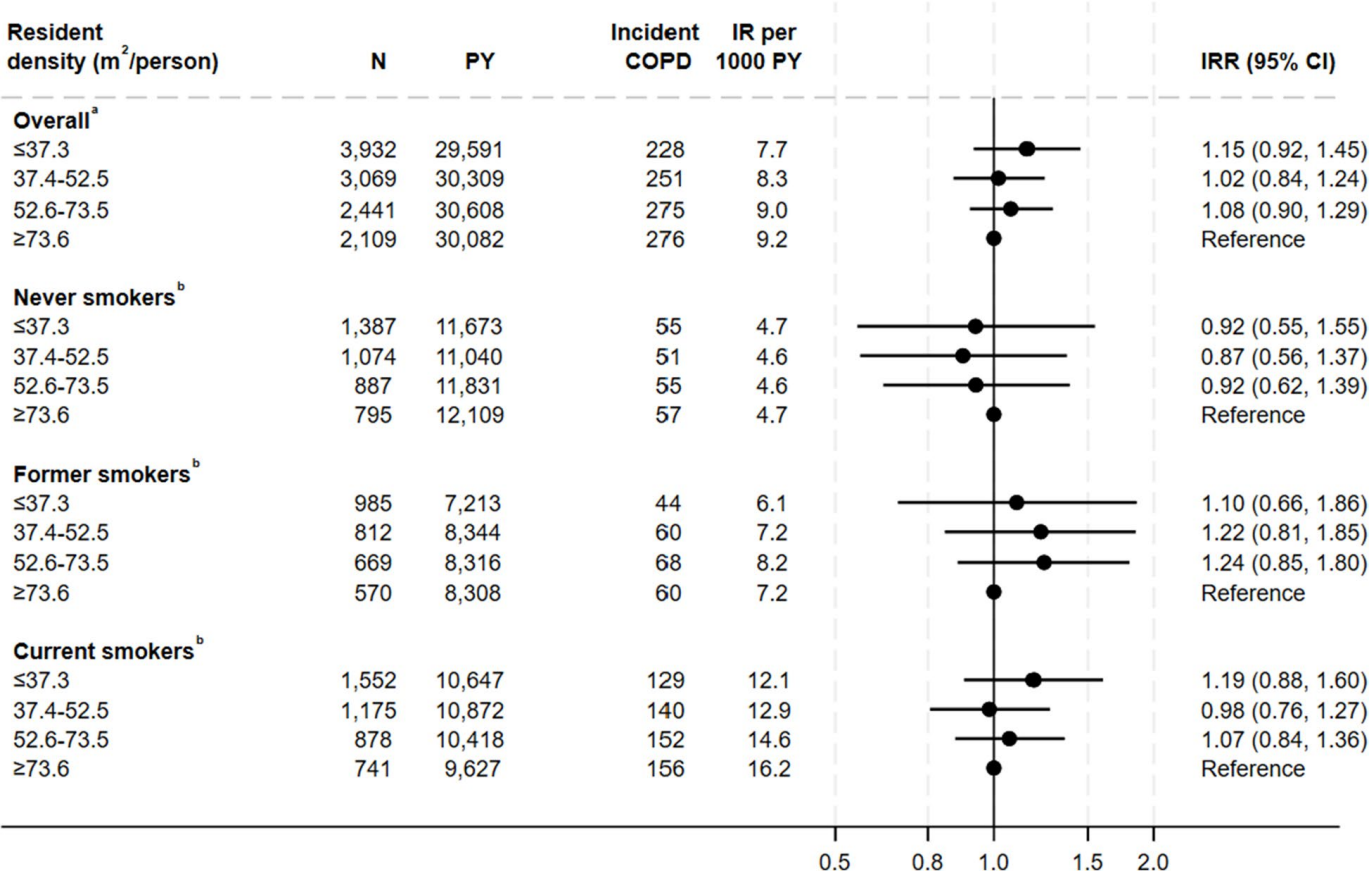
Association between residence density and incidence rate of Chronic Obstructive Pulmonary Disease ^a^Adjusted for year of construction, urbanization, type of housing, educational level, household income, age, sex, cohabitation, smoking status, and calendar year. Weighted for non-response. ^b^Adjusted for year of construction, urbanization, type of housing, educational level, household income, age, sex, cohabitation, and calendar year. Weighted for non-response COPD Chronic Obstructive Pulmonary Disease, CI confidence interval, IR Incidence rate, IRR Incidence rate ratio, PY Person years

Individuals living in more dense households had a tendency of a higher risk of COPD with an IRR of 1.15 (95% CI 0.92–1.45) compared to individuals living in the least dense households. When analysis was stratified by smoking status, this pattern was only seen among former and current smokers (Fig. 6).

### Association between perceived indoor environment and COPD

**Fig. 7:**
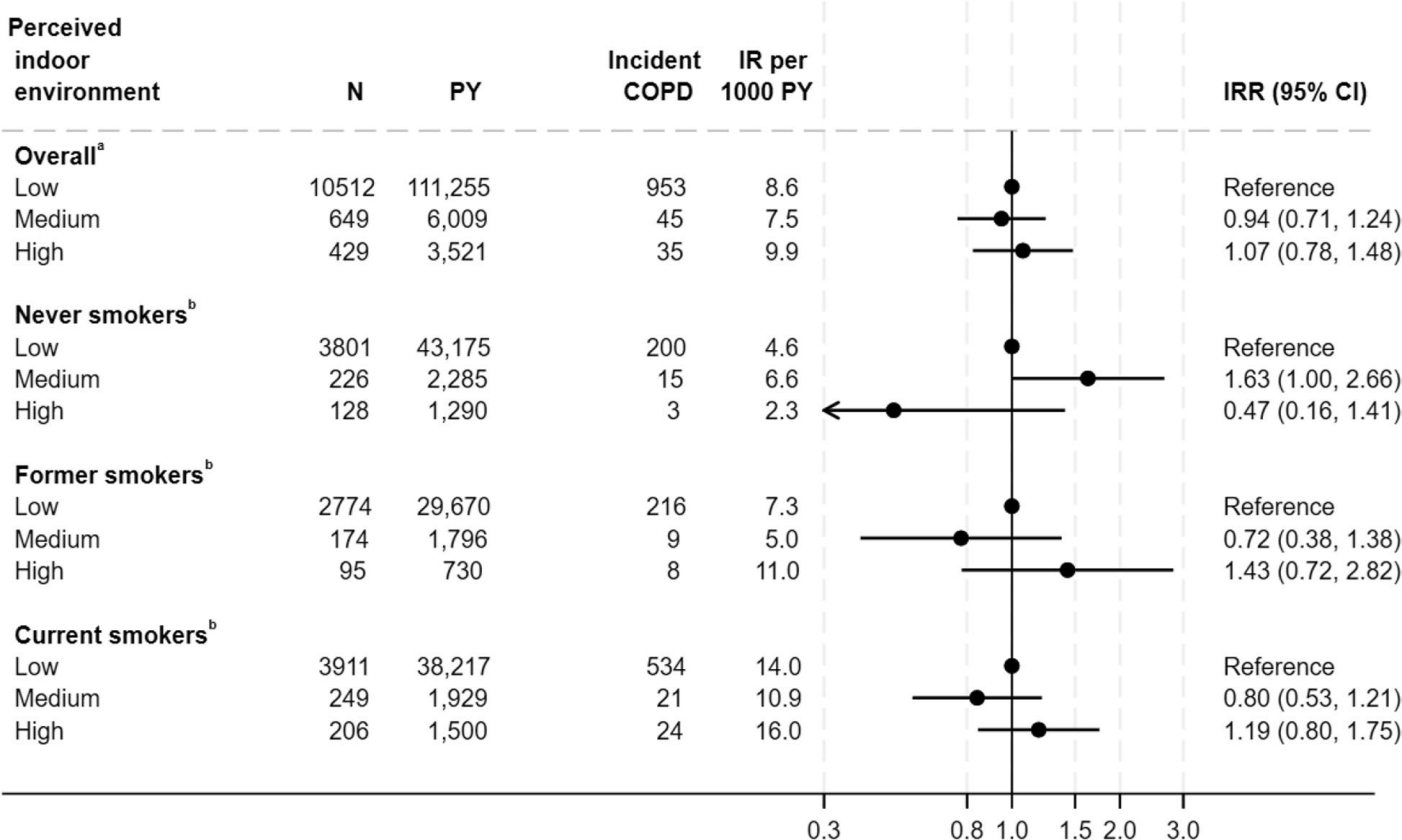
Association between perceived indoor environment (low, medium, and high level of annoyances) and the incidence rate of Chronic Obstructive Pulmonary Disease ^a^Adjusted for year of construction, urbanization, home ownership, resident density, educational level, household income, age, sex, cohabitation, smoking status, calendar year, and time lived in residence. Weighted for non-response. ^b^Adjusted for year of construction, urbanization, home ownership, resident density, educational level, household income, age, sex, cohabitation, calendar year, and time lived in residence. Weighted for non-response COPD Chronic Obstructive Pulmonary Disease, CI confidence interval, IR Incidence rate, IRR Incidence rate ratio, PY Person years

There was no association between perceived indoor environment and risk of COPD the overall analysis. However, when stratified by smoking status the pattern differed between never smokers and former/current smokers. The IR of COPD was 63% higher among never smokers reporting medium level of annoyances compared to individuals with low level of annoyances, while the IR was lower among former/current smokers. (Fig. 7).

### Sensitivity analyses

Similar results were seen between resident density or perceived indoor environment and risk of COPD, regardless of whether adjustments were made for type of housing or home ownership (data not shown).

## Discussion

In this cohort study with up to 19 years of follow-up, we found that individuals living outside the biggest cities had a lower risk of developing COPD compared to individuals living in larger cities. In contrast, individuals living in semi-detached houses, and individuals living in rented homes had a higher risk of developing COPD compared to individuals living in detached houses and owned homes, respectively. Also, we did find lower IR of COPD among individuals living in dwellings built after 1983 compared to before 1962 and higher IR of COPD among individuals living in the densest households, not statistically significant, though. Overall, similar patterns were seen when stratified by smoking status, however, the examined exposure tended to influence smokers slightly less. Only for perceived indoor environment opposite patterns were seen, but estimates were uncertain.

### Interpretation of findings

Individuals living in dwellings built after 1982 may have a lower risk compared to individuals living in dwellings built before 1962. This association might be explained by factors related to the construction year. As an example, previous Danish research has shown that individuals living in older dwellings were more likely to have mold and moisture, water damage, lack of exhaust hood use with the use of a gas stove and use of fireplace [32]. Therefore, the lower risk of COPD among individuals living in dwellings built in or after 1982 compared to individuals living in dwellings built before 1962 might partly be due to differences in exposure to mold, moisture, particle pollution etc.

A lower risk of developing COPD among individuals living outside larger cities is in line with recent estimates from a systematic review and modelling analysis of risk factors for COPD [26]. The association might be due to a lower exposure to air pollution, as PM_2.5_ and NO_2_ recently have been associated with the risk of developing COPD in a systematic review and meta-analysis [4].

Individuals living in apartments and semi-detached houses had a higher risk of developing COPD. Type of housing has previously been associated with the risk of COPD in a cross-sectional study by Gan et al. [13] who found that individuals living in mobile homes had a higher risk of COPD. However, due to the cross-sectional design, it was not possible to disentangle the time relation between the choice of home and development of disease since individuals with exiting COPD might choose a more COPD-friendly home (e.g., without stairs). In our study we can exclude this as a possible explanation since individuals were free of COPD at study inclusion (i.e., also when moving into the home studied). Socioeconomic factors may play a key role when selecting which housing type to live in [13]. Moreover, socioeconomic position is a known risk factor for COPD [26]. Therefore, socioeconomic position was a major confounding factor in the association between type of housing and COPD. To address this, analyses were adjusted for both educational level and household income, however, we cannot exclude the possibility that some residual confounding of socioeconomic position might still exist. Home ownership can also be regarded as a marker of socioeconomic position [13, 28]. In the current study, we could not adjust the association between type of housing and COPD for home ownership as the overlap between ownership and type of housing was too large in the dataset (94% of individuals living in detached houses owned their house and 85% of individuals living in apartments rented their home). Yet, home ownership might reflect both socioeconomic position [13, 28] and the possibility for homeowners to change undesirable home conditions that can affect their health [33]. Therefore, home ownership was not used as a marker of socioeconomic position but investigated as a separate exposure for the risk of COPD.

Individuals living in rented homes had a higher risk of COPD compared to individuals living in owned homes. Previous research has found an association between home ownership and respiratory health [11]. The association might be complex and work through several mechanisms [33], however, the most important for respiratory health is probably the ability for home owners to make structural changes or adjustments to their dwellings which can improve housing quality (e.g., avoid moisture, mold and draught) and thereby health. We found an increased risk of COPD among individuals living in semidetached houses and apartments as well as among individuals living in rented homes. However, there was a large overlap between the type of housing and home ownership, thus these two factors might reflect the same underlying mechanism. Further research with the power to analyze home ownership stratified by housing type can help shed light on this interrelationship.

There was no difference in risk of COPD between individuals living in more dense residents compared to individuals living in less dense residences. Household density has been associated with higher levels of particulate matter [34] and mold [32] Therefore, we hypothesized that individuals living in denser households would have a higher risk of COPD. Our data did not support such an association. However, among smokers, the risk was higher for individuals in the densest residences (≤ 37.3 m^2^ per person), indicating that living crowded is worse among smokers. This needs further investigation. Behaviors such as cleaning and ventilation can also be relevant in the context of resident density but were beyond the scope of this study.

Individuals reporting moderate and many annoyances in the indoor environment did not have a higher risk of COPD compared to individuals reporting few annoyances. This may have several explanations. First and foremost, the reported annoyances are diverse and not all of them might have an impact on the etiology of COPD, e.g., noise from neighbors. Among the individuals with the most annoyances, dominating annoyances were temperature and draught [19] which previously have been associated to respiratory health [11]. Therefore, an association between the group of individuals reporting most annoyances and COPD is plausible. However, when analyzing the association between each of the reported annoyances and COPD, no association was found either (data not shown). Therefore, it may be that the questions were not framed to catch, e.g., mold exposure precisely enough to be able to associate mold with the risk of COPD. Nevertheless, analyzing separate exposures in the home environment should always be done with caution since people are exposed to several indoor exposures simultaneously, of which several will correlate [35, 36]. Therefore, it is difficult to disentangle whether it is the presence of the exposure or the absence of another that is responsible for the association. The presence of exposures might also strengthen each other, or the accumulation of several exposures might be the triggering factor. This may also explain why different patterns were seen among never smokers and former/current smokers. However, more research is needed in both specific indoor exposures as well as the combination of exposures in the indoor environment and the risk of COPD.

For most of the housing conditions, with exception of perceived indoor environment, similar patterns were seen among never smokers, former smokers, and current smokers. However, current smokers seemed to be less influenced.

All studied exposures in the current study are to some degree interrelated or on the same causal path between housing conditions and COPD. Mediation analyses that can help disentangle the contribution of each factor to the overall risk is of importance in future research.

### Strength and limitations

A major strength of the study is the cohort design where we follow individuals free of COPD for the development of incident COPD and the long follow-up allowed by the Danish nationwide registers. Furthermore, COPD was defined using register-based data and therefore not prone to recall bias as opposed to using self-reported information. COPD diagnoses in the Danish National Patient Register has shown to have a high positive predictive value (92%) [37].

Our study is based on a representative random sample of individuals above 30 years of age which allows us to generalize the findings to the general Danish population. The participation rate was high (74.2%), however, participants and non-participants may differ and therefore analyses were weighted for non-participation minimizing the influence of selection bias. A further strength of the study is that we included a unique set of confounders for each studied exposure, thereby minimizing the risk of confounding or, e.g., controlling for a mediator (a concept known as Table 2 Fallacy [38, 39]).

A key limitation of our study is the risk of residual confounding by socioeconomic position. Socioeconomic differences in the risk of, e.g., hospitalization for COPD is seen among both men and women and at all ages [40]. We assessed socioeconomic position by both highest attained educational level and household income. The latter was assessed yearly. Since the population is ≥ 30 years, the highest attained educational level is assumed to be stable, thereby we include both a stable measure (education) and a time-varying measure (household income). However, since socioeconomic position is often assessed by housing condition (e.g., home ownership) [28], it is difficult to disentangle the contribution from housing condition and the contribution from other socioeconomic risk factors. Another limitation of our study is that we do not have any information about occupational exposure to dust or smoke, which is a known risk factor for COPD [26]. However, since occupational exposure to dust and smoke are not associated with the studied housing condition, occupational exposure is not a confounder in the studied associations. Occupational exposures are partly associated with socioeconomic position (see DAGs in supplementary), with lower socioeconomic position being associated with higher exposure to dust and smoke [41]. The analyses are adjusted for socioeconomic position, thus occupational exposure to dust and smoke are to some degree accounted for. Lastly, in the stratified analyses the power was low resulting in wide confidence intervals. However, the analyses contributed with suggestive information on whether patterns were similar among smokers and non-smokers.

## Conclusion

In this cohort study with up to 19 years of follow-up, we found that individuals living outside the biggest cities had a lower risk of developing COPD compared to individuals living in larger cities. In contrast, individuals living in semi-detached houses or living in rented homes had a higher risk of COPD compared to individuals living in detached houses or owned homes, respectively. Living in newer dwellings (built after 1982) may lower the risk of COPD while living in the most densest household may increase the risk. No association was seen between perceived indoor environment and risk of COPD.

### Supplementary Information

Additional file 1. Table S1. ICD-8^1^, ICD-10 and ATC codes used to define COPD. Table S2a. Baseline characteristics of the study population according to year of construction (*n*=11,557). Table S2b.Baseline characteristics of the study population according to urbanization (*n*=11,590). Table S2c. Baseline characteristics of the study population according to type of housing (*n*=11,517). Table S2d. Baseline characteristics of the study population according to resident density (*n*=11,551). Table S2e. Baseline characteristics of the study population according to perceived indoor environment (*n*=11,590). Figure S1a. Directed Acyclic Graph (DAG) of the association between year of construction and Chronic Obstructive Pulmonary Disease (COPD). Figure S1b. Directed Acyclic Graph (DAG) of the association between urbanization and Chronic Obstructive Pulmonary Disease (COPD). Figure S1c. Directed Acyclic Graph (DAG) of the association between type of housing and Chronic Obstructive Pulmonary Disease (COPD). Figure S1d. Directed Acyclic Graph (DAG) of the association between home ownership and Chronic Obstructive Pulmonary Disease (COPD). Figure S1e. Directed Acyclic Graph (DAG) of the association between resident density and Chronic Obstructive Pulmonary Disease (COPD). Figure S1f. Directed Acyclic Graph (DAG) of the association between perceived indoor environment and Chronic Obstructive Pulmonary Disease (COPD).

## Data Availability

The datasets generated and/or analyzed during the current study are not publicly available due to regulations formulated by the Danish Data Protection Law and Statistics Denmark (data are located on a secure server at Statistics Denmark). Access to data can only be granted to researchers in Danish research environments after approval from the Danish Data Protection Agency and Statistics Denmark, respectively.

## ABBREVIATIONS

ATC: Anatomical Therapeutic Chemical
BMI: Body Mass Index
CI: Confidence Interval
COPD: Chronic Obstructive Pulmonary Disease
DAG: Directed acyclic graph
IQR: Interquartile range
IR: Incidence rate
IRR: Incidence rate ratio
ISCED: International Standard Classification of Education
PY: Person Years
