# Supplementary material for: Housing conditions and risk of incident COPD: a Danish cohort study, 2000–2018
Source: BMC Public Health. 2024 Jun 27;24:1714. doi: 10.1186/s12889-024-19131-3 (PMC11210200; doi:10.1186/s12889-024-19131-3)
Supplement: Supplementary file 1 — Additional file 1. Table S1. ICD-81, ICD-10 and ATC codes used to define COPD. Table S2a. Baseline characteristics of the study population according to year of construction (n=11,557). Table S2b.Baseline characteristics of the study population according to urbanization (n=11,590). Table S2c. Baseline characteristics of the study population according to type of housing (n=11,517). Table S2d. Baseline characteristics of the study population according to resident density (n=11,551). Table S2e. Baseline characteristics of the study population according to perceived indoor environment (n=11,590). Figure S1a. Directed Acyclic Graph (DAG) of the association between year of construction and Chronic Obstructive Pulmonary Disease (COPD). Figure S1b. Directed Acyclic Graph (DAG) of the association between urbanization and Chronic Obstructive Pulmonary Disease (COPD). Figure S1c. Directed Acyclic Graph (DAG) of the association between type of housing and Chronic Obstructive Pulmonary Disease (COPD). Figure S1d. Directed Acyclic Graph (DAG) of the association between home ownership and Chronic Obstructive Pulmonary Disease (COPD). Figure S1e. Directed Acyclic Graph (DAG) of the association between resident density and Chronic Obstructive Pulmonary Disease (COPD). Figure S1f. Directed Acyclic Graph (DAG) of the association between perceived indoor environment and Chronic Obstructive Pulmonary Disease (COPD). [file 12889_2024_19131_MOESM1_ESM.docx]

**Supplementary material for:**

**Housing condition and risk of incident COPD: A Danish cohort study, 2000-2018**

**Table S1.** ICD-8^1^, ICD-10 and ATC codes used to define COPD.

| **ICD-8** | **ICD-10** | **ATC** |
| --- | --- | --- |
| 490-492 | J44 as primary diagnosis  J96 as primary diagnosis in combination with J44 as secondary diagnosis  J13-J18 as primary diagnosis in combination with J44 or J96 as one of the secondary diagnoses | All redeemed prescriptions with an indication code 379 or 464 or an ATC code R03A or R03B  Prescriptions were only included if at least two prescriptions within a 12-month period were redeemed. |
| ^1^ICD-8 codes were used to identify prevalent COPD.  To identify individuals with existing COPD (prevalent) information from the Danish National Patient Register 10 years before the date of interview was used.  ICD-8: International Classification of Diseases, Eighth Revision  ICD-10: International Classification of Diseases and Related Health Problems, Tenth Revision  ATC: Anatomic Therapeutic chemical classification | | |

**Table S2a**. Baseline characteristics of the study population according to year of construction (n=11,557).

|  | **Year of Construction** | | | | | |
| --- | --- | --- | --- | --- | --- | --- |
|  | **<1962** | | **1962-1982** | | **≥1983** | |
|  | **N** | **%** | **N** | **%** | **N** | **%** |
|  |  |  |  |  |  |  |
| **Sex** |  |  |  |  |  |  |
| Men | 2,817 | 50.6 | 2,189 | 48.8 | 702 | 46.6 |
| Women | 2,745 | 49.4 | 2,301 | 51.2 | 803 | 53.4 |
|  |  |  |  |  |  |  |
| **Age (years)** |  |  |  |  |  |  |
| 30-39 | 1,433 | 25.8 | 827 | 18.4 | 429 | 28.5 |
| 40-49 | 1,301 | 23.4 | 1,044 | 23.3 | 365 | 24.3 |
| 50-59 | 1,181 | 21.2 | 1,291 | 28.8 | 236 | 15.7 |
| 60-69 | 732 | 13.2 | 749 | 16.7 | 177 | 11.8 |
| 70-79 | 602 | 10.8 | 372 | 8.3 | 177 | 11.8 |
| ≥80 | 313 | 5.6 | 207 | 4.6 | 121 | 8.0 |
|  |  |  |  |  |  |  |
| **Cohabitation** |  |  |  |  |  |  |
| Cohabitating | 3,898 | 70.1 | 3528 | 78.6 | 1026 | 68.2 |
| Living alone | 1,664 | 29.9 | 962 | 21.4 | 479 | 31.8 |
|  |  |  |  |  |  |  |
| **Educational level** |  |  |  |  |  |  |
| Elementary | 2,007 | 36.1 | 1,535 | 34.2 | 527 | 35.0 |
| Short | 2,220 | 39.9 | 1,949 | 43.5 | 654 | 43.5 |
| Medium/long | 1,333 | 24.0 | 1,101 | 22.3 | 323 | 21.5 |
|  |  |  |  |  |  |  |
| **Household income** |  |  |  |  |  |  |
| Lowest quintile | 1,283 | 23.1 | 670 | 14.9 | 355 | 23.6 |
| Second quintile | 1,214 | 21.8 | 804 | 17.9 | 289 | 19.2 |
| Third quintile | 1,118 | 20.1 | 898 | 20.0 | 294 | 19.5 |
| Fourth quintile | 1,036 | 18.6 | 981 | 21.8 | 303 | 20.1 |
| Highest quintile | 911 | 16.4 | 1,137 | 25.3 | 264 | 17.5 |
|  |  |  |  |  |  |  |
| **Body Mass Index (kg/m^2^)** |  |  |  |  |  |  |
| <18.5 | 120 | 2.2 | 100 | 2.3 | 31 | 2.1 |
| 18.5-24.9 | 2,791 | 51.1 | 2,153 | 48.9 | 804 | 54.4 |
| 25-29.9 | 1,972 | 36.1 | 1,676 | 38.0 | 505 | 34.1 |
| ≥30 | 580 | 10.6 | 478 | 10.8 | 139 | 9.4 |
|  |  |  |  |  |  |  |
| **Smoking** |  |  |  |  |  |  |
| Never | 1,937 | 34.9 | 1,646 | 36.8 | 563 | 37.5 |
| Former | 1,398 | 25.2 | 1,229 | 27.5 | 409 | 27.2 |
| Current | 2,219 | 40.0 | 1,600 | 35.8 | 530 | 35.3 |
|  |  |  |  |  |  |  |
| **Time lived in residence (years)** |  |  |  |  |  |  |
| <3 | 1,157 | 20.8 | 720 | 16.0 | 505 | 33.6 |
| 3-10 | 1,782 | 32.0 | 1,228 | 27.3 | 682 | 45.3 |
| 11-20 | 1,123 | 20.2 | 1,029 | 22.9 | 304 | 20.2 |
| 21 | 1,500 | 27.0 | 1,513 | 33.7 | 14 | 0.9 |

**Table S2b.** Baseline characteristics of the study population according to urbanization (n=11,590).

|  | **Urbanization** | | | | | | | |
| --- | --- | --- | --- | --- | --- | --- | --- | --- |
|  | **≥50.000** | | **5.000-49.999** | | **200-4.999** | | **rural** | |
|  | **N** | **%** | **N** | **%** | **N** | **%** | **N** | **%** |
|  |  |  |  |  |  |  |  |  |
| **Sex** |  |  |  |  |  |  |  |  |
| Men | 1,340 | 48.6 | 1,743 | 48.9 | 1,597 | 49.4 | 1,048 | 51.5 |
| Women | 1,418 | 51.4 | 1,819 | 51.1 | 1,639 | 50.6 | 986 | 48.5 |
|  |  |  |  |  |  |  |  |  |
| **Age (years)** |  |  |  |  |  |  |  |  |
| 30-39 | 749 | 27.2 | 795 | 22.3 | 651 | 20.1 | 501 | 24.6 |
| 40-49 | 591 | 21.4 | 802 | 22.5 | 802 | 24.8 | 521 | 25.6 |
| 50-59 | 583 | 21.1 | 880 | 24.7 | 768 | 23.7 | 483 | 23.7 |
| 60-69 | 350 | 12.7 | 523 | 14.7 | 469 | 14.5 | 317 | 15.6 |
| 70-79 | 315 | 11.4 | 353 | 9.9 | 344 | 10.6 | 139 | 6.8 |
| ≥80 | 170 | 6.2 | 209 | 5.9 | 202 | 6.2 | 73 | 3.6 |
|  |  |  |  |  |  |  |  |  |
| **Cohabitation** |  |  |  |  |  |  |  |  |
| Cohabitating | 1,753 | 63.6 | 2,528 | 71.0 | 2,510 | 77.6 | 1,674 | 82.3 |
| Living alone | 1,005 | 36.4 | 1,034 | 29.0 | 726 | 22.4 | 360 | 17.7 |
|  |  |  |  |  |  |  |  |  |
| **Educational level** |  |  |  |  |  |  |  |  |
| Elementary | 773 | 28.0 | 1,140 | 32.0 | 1,321 | 40.8 | 846 | 41.6 |
| Short | 1,172 | 42.6 | 1,577 | 44.3 | 1,315 | 40.7 | 770 | 37.9 |
| Medium/long | 809 | 29.4 | 843 | 23.7 | 599 | 18.5 | 417 | 20.5 |
|  |  |  |  |  |  |  |  |  |
| **Household income** |  |  |  |  |  |  |  |  |
| Lowest quintile | 543 | 19.7 | 609 | 17.1 | 686 | 21.2 | 488 | 24.0 |
| Second quintile | 536 | 19.4 | 637 | 17.9 | 649 | 20.1 | 491 | 24.1 |
| Third quintile | 520 | 18.9 | 694 | 19.5 | 685 | 21.2 | 414 | 20.4 |
| Fourth quintile | 566 | 20.5 | 790 | 22.2 | 650 | 20.1 | 316 | 15.5 |
| Highest quintile | 593 | 21.5 | 832 | 23.4 | 566 | 17.5 | 325 | 16.0 |
|  |  |  |  |  |  |  |  |  |
| **Body Mass Index (kg/m^2^)** |  |  |  |  |  |  |  |  |
| <18.5 | 70 | 2.6 | 88 | 2.5 | 62 | 2.0 | 34 | 1.7 |
| 18.5-24.9 | 1,493 | 55.0 | 1,797 | 51.4 | 1,515 | 48.0 | 955 | 47.5 |
| 25-29.9 | 905 | 33.3 | 1,281 | 36.6 | 1,195 | 37.9 | 782 | 38.9 |
| ≥30 | 246 | 9.1 | 332 | 9.5 | 384 | 12.2 | 239 | 11.9 |
|  |  |  |  |  |  |  |  |  |
| **Smoking** |  |  |  |  |  |  |  |  |
| Never | 927 | 33.7 | 1,283 | 36.1 | 1153 | 35.7 | 792 | 39.0 |
| Former | 757 | 27.5 | 946 | 26.6 | 842 | 26.1 | 498 | 24.5 |
| Current | 1,066 | 38.8 | 1,325 | 37.3 | 1234 | 38.2 | 741 | 36.5 |
|  |  |  |  |  |  |  |  |  |
| **Time lived in residence (years)** |  |  |  |  |  |  |  |  |
| <3 | 610 | 22.1 | 803 | 22.5 | 653 | 20.2 | 337 | 16.6 |
| 3-10 | 962 | 34.9 | 1,184 | 33.2 | 916 | 28.3 | 639 | 31.4 |
| 11-20 | 534 | 19.4 | 712 | 20.0 | 739 | 22.8 | 473 | 23.3 |
| 21 | 652 | 23.6 | 863 | 24.2 | 928 | 28.7 | 585 | 28.8 |

**Table S2c.** Baseline characteristics of the study population according to type of housing (n=11,517).

|  | **Type of housing** | | | | | | | | | |
| --- | --- | --- | --- | --- | --- | --- | --- | --- | --- | --- |
|  | **Detached house** | | **Semi-detached** | | **Apartment** | | **Farm** | | **Other** | |
|  | **N** | **%** | **N** | **%** | **N** | **%** | **N** | **%** | **N** | **%** |
|  |  |  |  |  |  |  |  |  |  |  |
| **Sex** |  |  |  |  |  |  |  |  |  |  |
| Men | 3,277 | 50.8 | 862 | 45.5 | 879 | 45.3 | 523 | 52.8 | 139 | 56.5 |
| Women | 3,168 | 49.2 | 1,034 | 54.5 | 1,061 | 54.7 | 467 | 47.2 | 107 | 43.5 |
|  |  |  |  |  |  |  |  |  |  |  |
| **Age (years)** |  |  |  |  |  |  |  |  |  |  |
| 30-39 | 1,433 | 22.2 | 406 | 21.4 | 555 | 28.6 | 255 | 25.8 | 27 | 11.0 |
| 40-49 | 1,650 | 25.6 | 449 | 23.7 | 318 | 16.4 | 247 | 24.9 | 36 | 14.6 |
| 50-59 | 1,673 | 26.0 | 391 | 20.6 | 360 | 18.6 | 244 | 24.6 | 33 | 13.4 |
| 60-69 | 949 | 14.7 | 261 | 13.8 | 267 | 13.8 | 142 | 14.3 | 31 | 12.6 |
| 70-79 | 521 | 8.1 | 249 | 13.1 | 278 | 14.3 | 70 | 7.1 | 24 | 9.8 |
| ≥80 | 219 | 3.4 | 140 | 7.4 | 162 | 8.4 | 32 | 3.2 | 95 | 38.6 |
|  |  |  |  |  |  |  |  |  |  |  |
| **Cohabitation** |  |  |  |  |  |  |  |  |  |  |
| Cohabitating | 5,501 | 85.4 | 1,147 | 60.5 | 844 | 43.5 | 843 | 85.2 | 84 | 34.1 |
| Living alone | 944 | 14.6 | 749 | 39.5 | 1,096 | 56.5 | 147 | 14.8 | 162 | 65.9 |
|  |  |  |  |  |  |  |  |  |  |  |
| **Educational level** |  |  |  |  |  |  |  |  |  |  |
| Elementary | 2,068 | 32.1 | 730 | 38.5 | 715 | 37.0 | 424 | 42.9 | 122 | 49.8 |
| Short | 2,829 | 43.9 | 733 | 38.7 | 769 | 39.7 | 374 | 37.8 | 93 | 38.0 |
| Medium/long | 1,548 | 24.0 | 432 | 22.8 | 451 | 23.3 | 191 | 19.3 | 30 | 12.2 |
|  |  |  |  |  |  |  |  |  |  |  |
| **Household income** |  |  |  |  |  |  |  |  |  |  |
| Lowest quintile | 790 | 12.3 | 491 | 25.9 | 651 | 33.6 | 261 | 26.4 | 117 | 47.6 |
| Second quintile | 1,115 | 17.3 | 434 | 22.9 | 464 | 23.9 | 233 | 23.5 | 54 | 22.0 |
| Third quintile | 1,380 | 21.4 | 355 | 18.7 | 346 | 17.8 | 182 | 18.4 | 33 | 13.4 |
| Fourth quintile | 1,537 | 23.8 | 325 | 17.1 | 272 | 14.0 | 150 | 15.2 | 26 | 10.6 |
| Highest quintile | 1,623 | 25.2 | 291 | 15.3 | 207 | 10.7 | 164 | 16.6 | 16 | 6.5 |
|  |  |  |  |  |  |  |  |  |  |  |
| **Body Mass Index (kg/m^2^)** |  |  |  |  |  |  |  |  |  |  |
| <18.5 | 118 | 1.9 | 56 | 3.0 | 55 | 2.9 | 13 | 1.3 | 11 | 5.1 |
| 18.5-24.9 | 3,253 | 51.2 | 938 | 50.4 | 979 | 51.6 | 459 | 46.9 | 91 | 42.3 |
| 25-29.9 | 2,350 | 37.0 | 665 | 35.7 | 634 | 33.4 | 405 | 41.4 | 82 | 38.1 |
| ≥30 | 631 | 9.9 | 202 | 10.9 | 230 | 12.1 | 102 | 10.4 | 31 | 14.4 |
|  |  |  |  |  |  |  |  |  |  |  |
| **Smoking** |  |  |  |  |  |  |  |  |  |  |
| Never | 2,412 | 37.5 | 605 | 31.9 | 593 | 30.7 | 442 | 44.6 | 78 | 32.5 |
| Former | 1,759 | 27.3 | 500 | 26.4 | 456 | 23.6 | 239 | 24.1 | 69 | 28.8 |
| Current | 2,265 | 35.2 | 789 | 41.7 | 882 | 45.7 | 309 | 31.2 | 93 | 38.8 |
|  |  |  |  |  |  |  |  |  |  |  |
| **Time lived in residence (years)** |  |  |  |  |  |  |  |  |  |  |
| <3 | 1,022 | 15.9 | 547 | 28.9 | 568 | 29.3 | 128 | 12.9 | 121 | 49.2 |
| 3-10 | 1,862 | 28.9 | 701 | 37.0 | 737 | 38.0 | 295 | 29.8 | 82 | 33.3 |
| 11-20 | 1,502 | 23.3 | 377 | 19.9 | 314 | 16.2 | 225 | 22.7 | 27 | 11.0 |
| 21 | 2,059 | 31.9 | 271 | 14.3 | 321 | 16.5 | 342 | 34.5 | 16 | 6.5 |

**Table S2d.** Baseline characteristics of the study population according to resident density (n=11,551).

|  | **Quartiles of square meters per person (m^2^/person)** | | | | | | | |
| --- | --- | --- | --- | --- | --- | --- | --- | --- |
|  | **≤ 37.3 (1st)** | | **37.4-52.5 (2nd)** | | **52.6-73.5 (3rd)** | | **≥ 73.6 (4th)** | |
|  | **N** | **%** | **N** | **%** | **N** | **%** | **N** | **%** |
|  |  |  |  |  |  |  |  |  |
| **Sex** |  |  |  |  |  |  |  |  |
| Men | 1,504 | 51.9 | 1,434 | 49.6 | 1,418 | 48.8 | 1,348 | 47.2 |
| Women | 1,394 | 48.1 | 1,457 | 50.4 | 1,487 | 51.2 | 1,509 | 52.8 |
|  |  |  |  |  |  |  |  |  |
| **Age (years)** |  |  |  |  |  |  |  |  |
| 30-39 | 1,430 | 49.3 | 703 | 24.3 | 342 | 11.8 | 213 | 7.5 |
| 40-49 | 963 | 33.2 | 833 | 28.8 | 535 | 18.4 | 377 | 13.2 |
| 50-59 | 296 | 10.2 | 636 | 22.0 | 895 | 30.8 | 878 | 30.7 |
| 60-69 | 81 | 2.8 | 377 | 13.0 | 555 | 19.1 | 645 | 22.6 |
| 70-79 | 73 | 2.5 | 253 | 8.8 | 373 | 12.8 | 452 | 15.8 |
| ≥80 | 55 | 1.9 | 89 | 3.1 | 205 | 7.1 | 292 | 10.2 |
|  |  |  |  |  |  |  |  |  |
| **Cohabitation** |  |  |  |  |  |  |  |  |
| Cohabitating | 2,571 | 88.7 | 2452 | 84.8 | 2054 | 70.7 | 1372 | 48.0 |
| Living alone | 327 | 11.3 | 439 | 15.2 | 851 | 29.3 | 1485 | 52.0 |
|  |  |  |  |  |  |  |  |  |
| **Educational level** |  |  |  |  |  |  |  |  |
| Elementary | 859 | 29.6 | 953 | 33.0 | 1,141 | 39.3 | 1,115 | 39.1 |
| Short | 1,320 | 45.6 | 1,290 | 44.6 | 1,167 | 40.2 | 1,043 | 36.5 |
| Medium/long | 717 | 24.8 | 646 | 22.4 | 595 | 20.5 | 697 | 24.4 |
|  |  |  |  |  |  |  |  |  |
| **Household income** |  |  |  |  |  |  |  |  |
| Lowest quintile | 532 | 18.4 | 567 | 19.6 | 599 | 20.6 | 608 | 21.3 |
| Second quintile | 775 | 26.7 | 560 | 19.4 | 489 | 16.8 | 480 | 16.8 |
| Third quintile | 799 | 27.6 | 587 | 20.3 | 529 | 18.2 | 394 | 13.8 |
| Fourth quintile | 545 | 18.8 | 656 | 22.7 | 610 | 21.0 | 509 | 17.8 |
| Highest quintile | 247 | 8.5 | 521 | 18.0 | 678 | 23.3 | 866 | 30.3 |
|  |  |  |  |  |  |  |  |  |
| **Body Mass Index (kg/m^2^)** |  |  |  |  |  |  |  |  |
| <18.5 | 54 | 1.9 | 69 | 2.4 | 61 | 2.1 | 67 | 2.4 |
| 18.5-24.9 | 1,550 | 54.4 | 1,404 | 49.2 | 1,402 | 49.3 | 1,390 | 49.7 |
| 25-29.9 | 942 | 33.0 | 1,061 | 37.2 | 1,081 | 38.0 | 1,067 | 38.2 |
| ≥30 | 305 | 10.7 | 320 | 11.2 | 300 | 10.5 | 270 | 9.7 |
|  |  |  |  |  |  |  |  |  |
| **Smoking** |  |  |  |  |  |  |  |  |
| Never | 1,042 | 36.0 | 982 | 34.0 | 1,056 | 36.5 | 1,063 | 37.3 |
| Former | 695 | 24.0 | 766 | 26.5 | 785 | 27.1 | 790 | 27.7 |
| Current | 1,155 | 39.9 | 1138 | 39.4 | 1,055 | 36.4 | 998 | 35.0 |
|  |  |  |  |  |  |  |  |  |
| **Time lived in residence (years)** |  |  |  |  |  |  |  |  |
| <3 | 791 | 27.3 | 668 | 23.1 | 523 | 18.0 | 397 | 13.9 |
| 3-10 | 1,209 | 41.7 | 976 | 33.8 | 814 | 28.0 | 692 | 24.2 |
| 11-20 | 669 | 23.1 | 640 | 22.1 | 586 | 20.2 | 559 | 19.6 |
| 21 | 229 | 7.9 | 607 | 21.0 | 982 | 33.8 | 1,209 | 42.3 |

**Table S2e.** Baseline characteristics of the study population according to perceived indoor environment (n=11.590).

|  | **Perceived indoor environment** | | | | | |
| --- | --- | --- | --- | --- | --- | --- |
|  | **Low** | | **Medium** | | **High** | |
|  | **N** | **%** | **N** | **%** | **N** | **%** |
|  |  |  |  |  |  |  |
| **Sex** |  |  |  |  |  |  |
| Men | 5,215 | 49.6 | 330 | 50.8 | 183 | 42.7 |
| Women | 5,297 | 50.4 | 319 | 49.2 | 246 | 57.3 |
|  |  |  |  |  |  |  |
| **Age (years)** |  |  |  |  |  |  |
| 30-39 | 2,349 | 22.3 | 174 | 26.8 | 173 | 40.3 |
| 40-49 | 2,451 | 23.3 | 169 | 26.0 | 96 | 22.4 |
| 50-59 | 2,504 | 23.8 | 144 | 22.2 | 66 | 15.4 |
| 60-69 | 1,523 | 14.5 | 85 | 13.1 | 51 | 11.9 |
| 70-79 | 1,072 | 10.2 | 50 | 7.7 | 29 | 6.8 |
| ≥80 | 613 | 5.8 | 27 | 4.2 | 14 | 3.3 |
|  |  |  |  |  |  |  |
| **Cohabitation** |  |  |  |  |  |  |
| Cohabitating | 7,746 | 73.7 | 445 | 68.6 | 274 | 63.9 |
| Living alone | 2,766 | 26.3 | 204 | 31.4 | 155 | 36.1 |
|  |  |  |  |  |  |  |
| **Educational level** |  |  |  |  |  |  |
| Elementary | 3,722 | 35.4 | 206 | 31.8 | 152 | 35.4 |
| Short | 4,372 | 41.6 | 299 | 46.1 | 163 | 38.0 |
| Medium/long | 2,411 | 23.0 | 143 | 22.1 | 114 | 26.6 |
|  |  |  |  |  |  |  |
| **Household income** |  |  |  |  |  |  |
| Lowest quintile | 2,084 | 19.8 | 123 | 19.0 | 119 | 27.7 |
| Second quintile | 2,082 | 19.8 | 133 | 20.5 | 98 | 22.8 |
| Third quintile | 2,076 | 19.7 | 139 | 21.4 | 98 | 22.8 |
| Fourth quintile | 2,119 | 20.2 | 140 | 21.6 | 63 | 14.7 |
| Highest quintile | 2,151 | 20.5 | 114 | 17.6 | 51 | 11.9 |
|  |  |  |  |  |  |  |
| **Body Mass Index (kg/m^2^)** |  |  |  |  |  |  |
| <18.5 | 222 | 2.2 | 14 | 2.2 | 18 | 4.2 |
| 18.5-24.9 | 5,201 | 50.4 | 340 | 53.5 | 219 | 51.7 |
| 25-29.9 | 3,800 | 36.8 | 212 | 33.3 | 151 | 35.6 |
| ≥30 | 1,095 | 10.6 | 70 | 11.0 | 36 | 8.5 |
|  |  |  |  |  |  |  |
| **Smoking** |  |  |  |  |  |  |
| Never | 3,801 | 36.2 | 226 | 34.8 | 128 | 29.8 |
| Former | 2,774 | 26.5 | 174 | 26.8 | 95 | 22.1 |
| Current | 3,911 | 37.3 | 249 | 38.4 | 206 | 48.0 |
|  |  |  |  |  |  |  |
| **Time lived in residence (years)** |  |  |  |  |  |  |
| <3 | 2,098 | 20.0 | 168 | 25.9 | 137 | 31.9 |
| 3-10 | 3,321 | 31.6 | 213 | 32.8 | 167 | 38.9 |
| 11-20 | 2,273 | 21.6 | 115 | 17.7 | 70 | 16.3 |
| 21 | 2,820 | 26.8 | 153 | 23.6 | 55 | 12.8 |

| **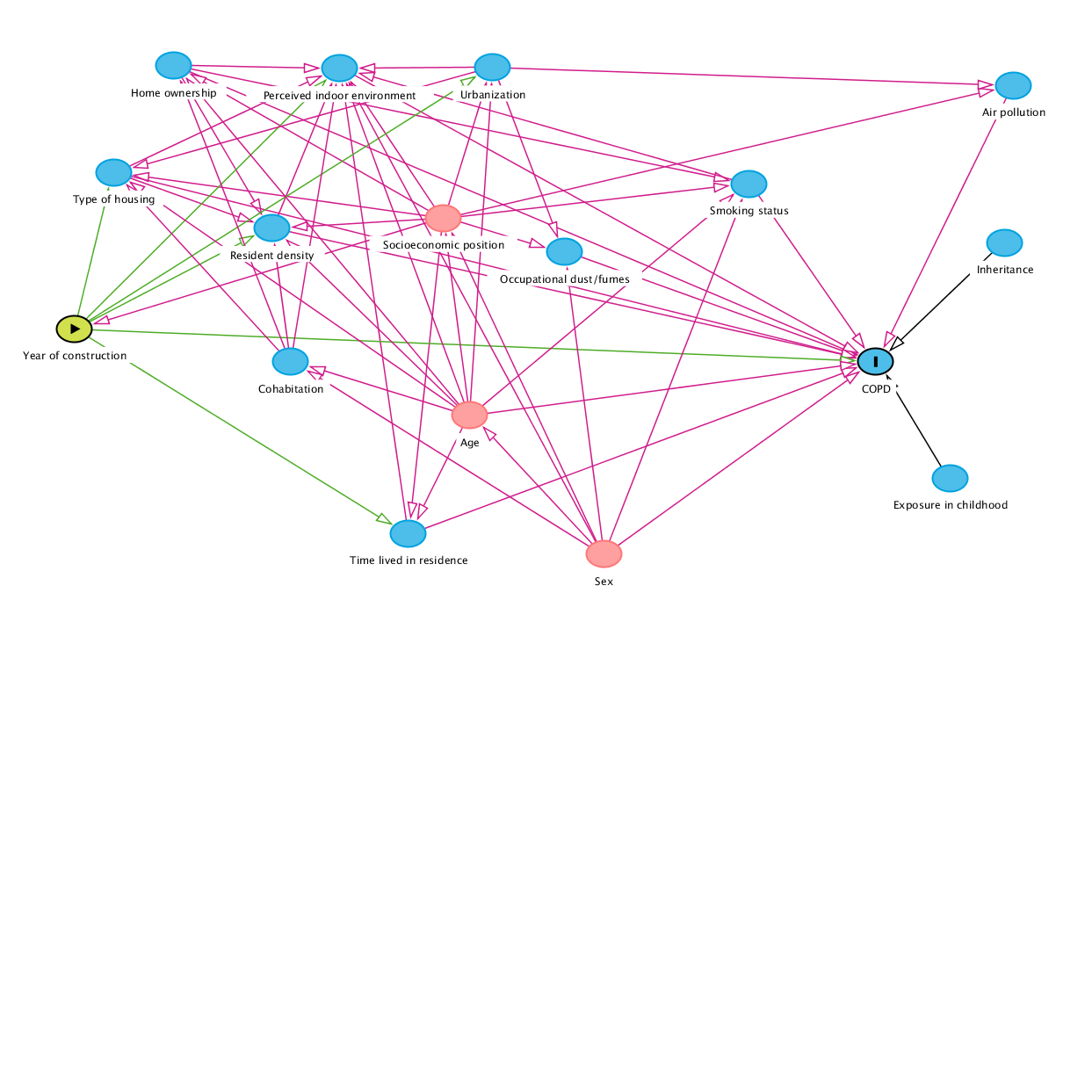**   |
| --- |
| **Figure S1a.** Directed Acyclic Graph (DAG) of the association between year of construction and Chronic Obstructive Pulmonary Disease (COPD). |

| **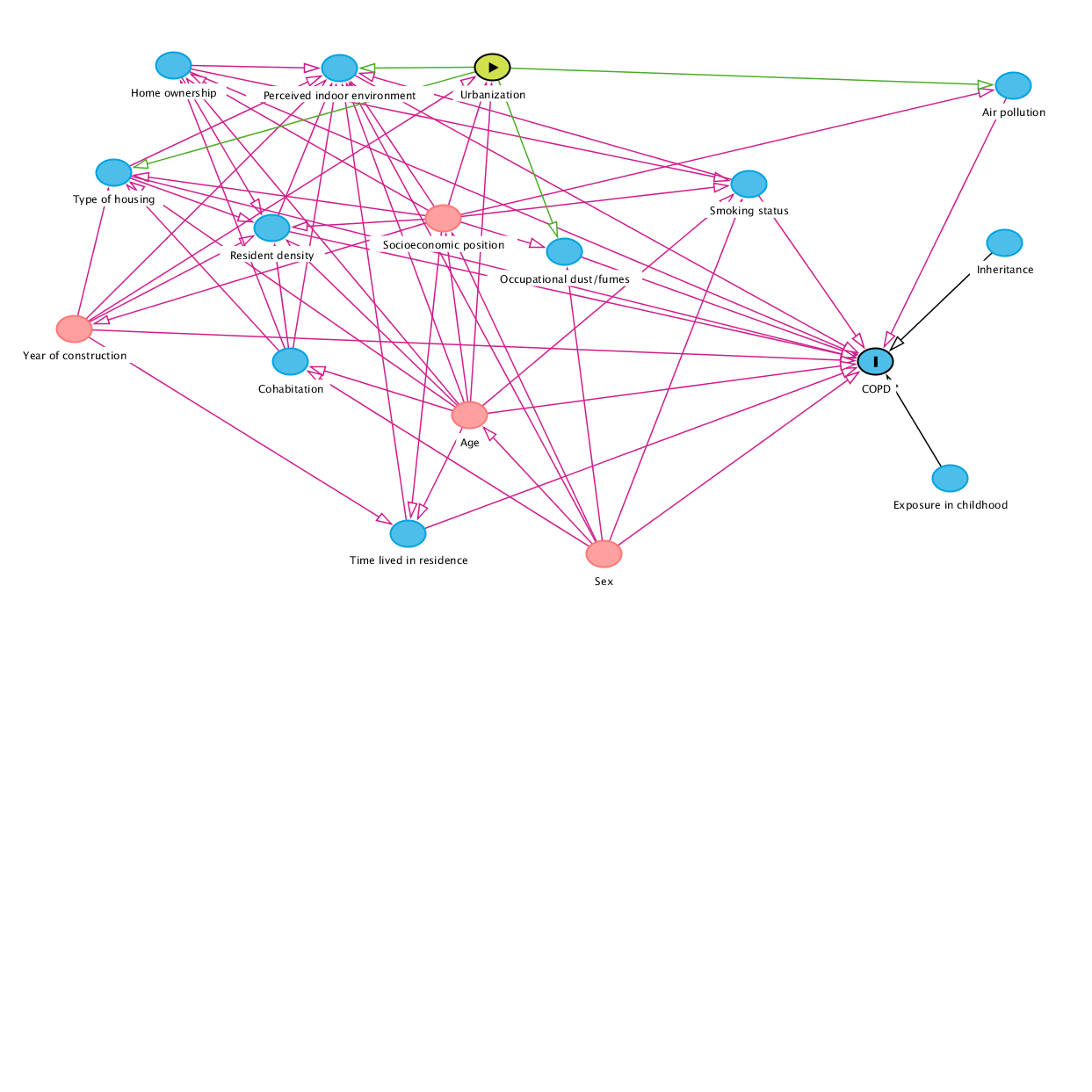**   |
| --- |
| **Figure S1b.** Directed Acyclic Graph (DAG) of the association between urbanization and Chronic Obstructive Pulmonary Disease (COPD). |
| **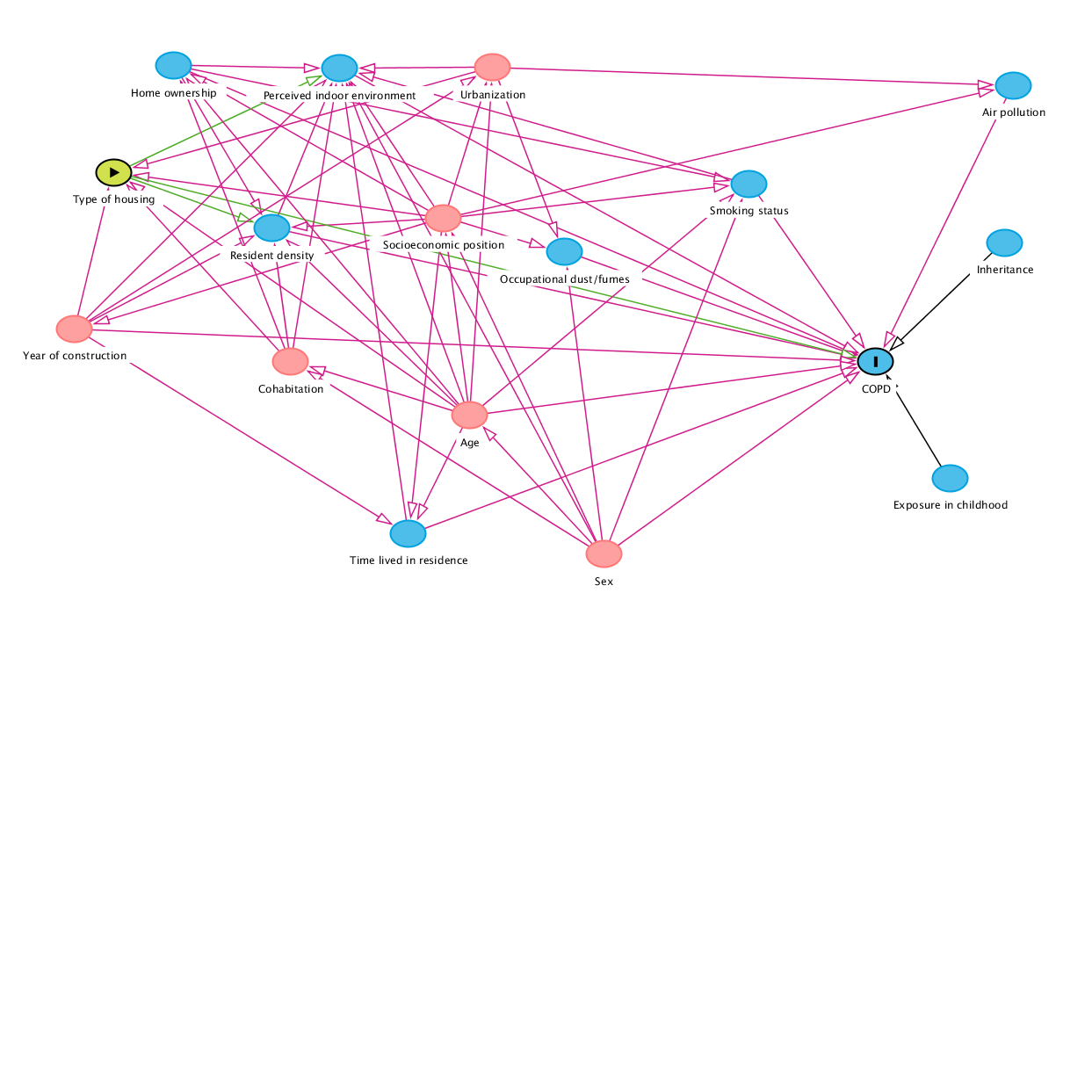**   |
| **Figure S1c.** Directed Acyclic Graph (DAG) of the association between type of housing and Chronic Obstructive Pulmonary Disease (COPD). |

| **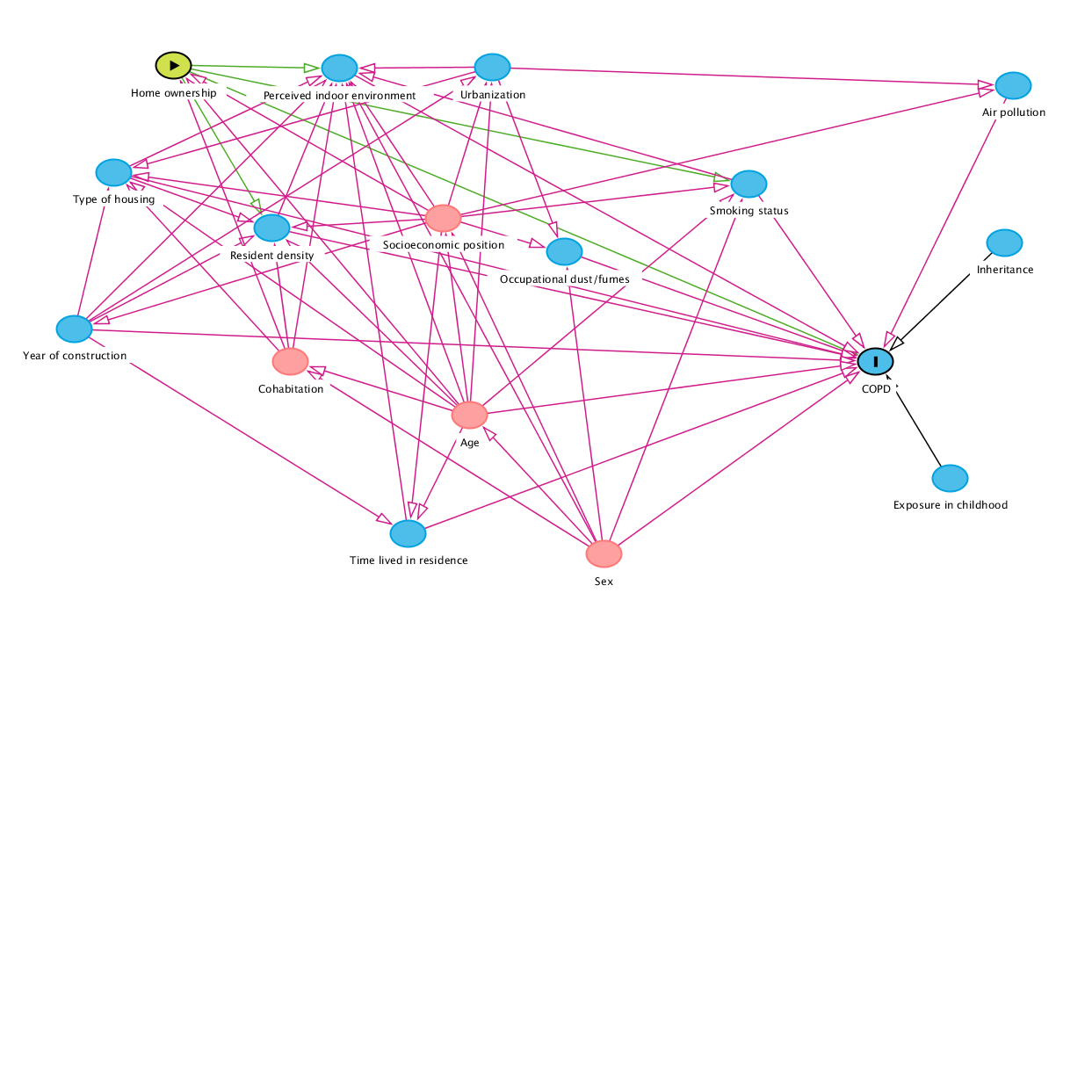**   |
| --- |
| **Figure S1d.** Directed Acyclic Graph (DAG) of the association between home ownership and Chronic Obstructive Pulmonary Disease (COPD). |

| **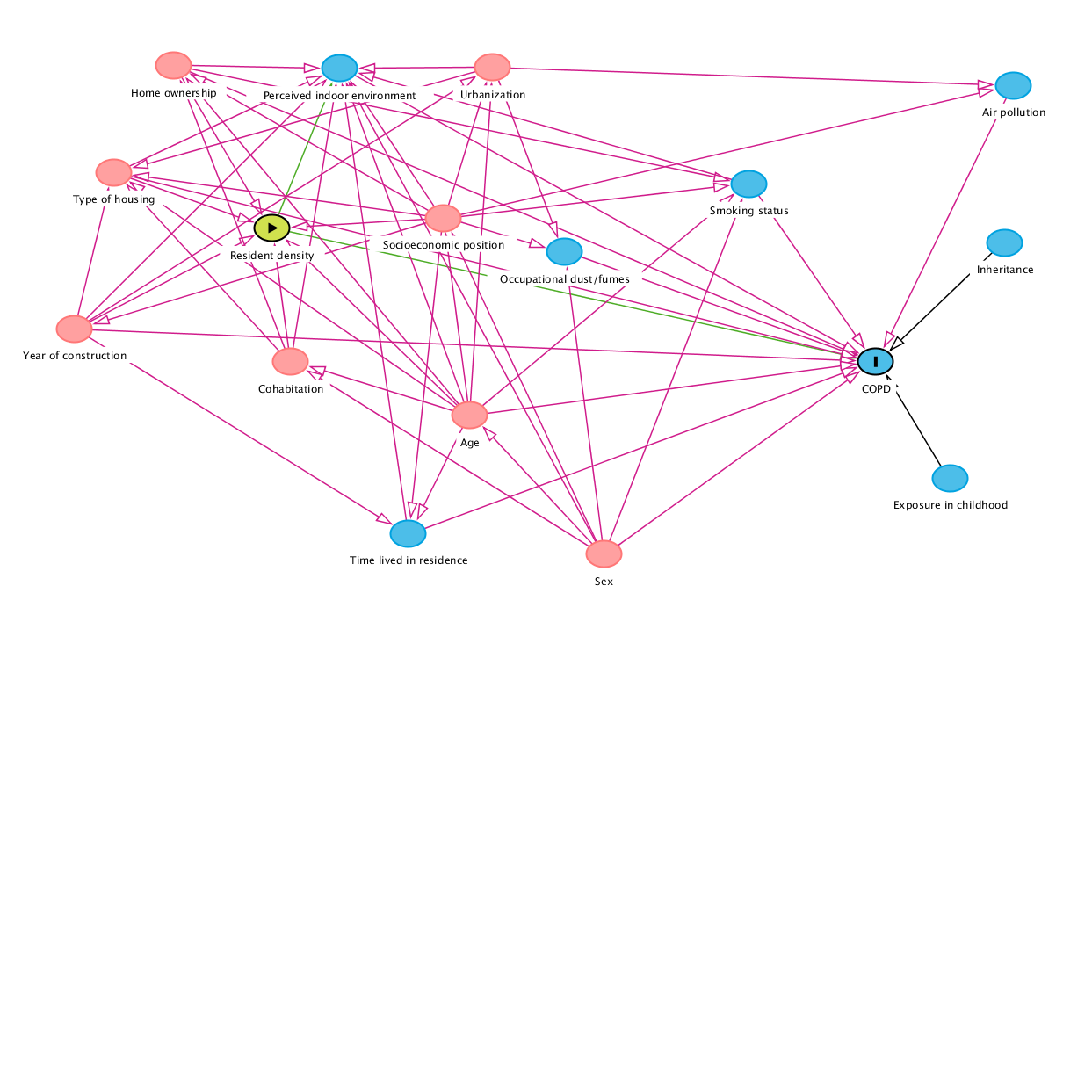**   |
| --- |
| **Figure S1e.** Directed Acyclic Graph (DAG) of the association between resident density and Chronic Obstructive Pulmonary Disease (COPD). |

| **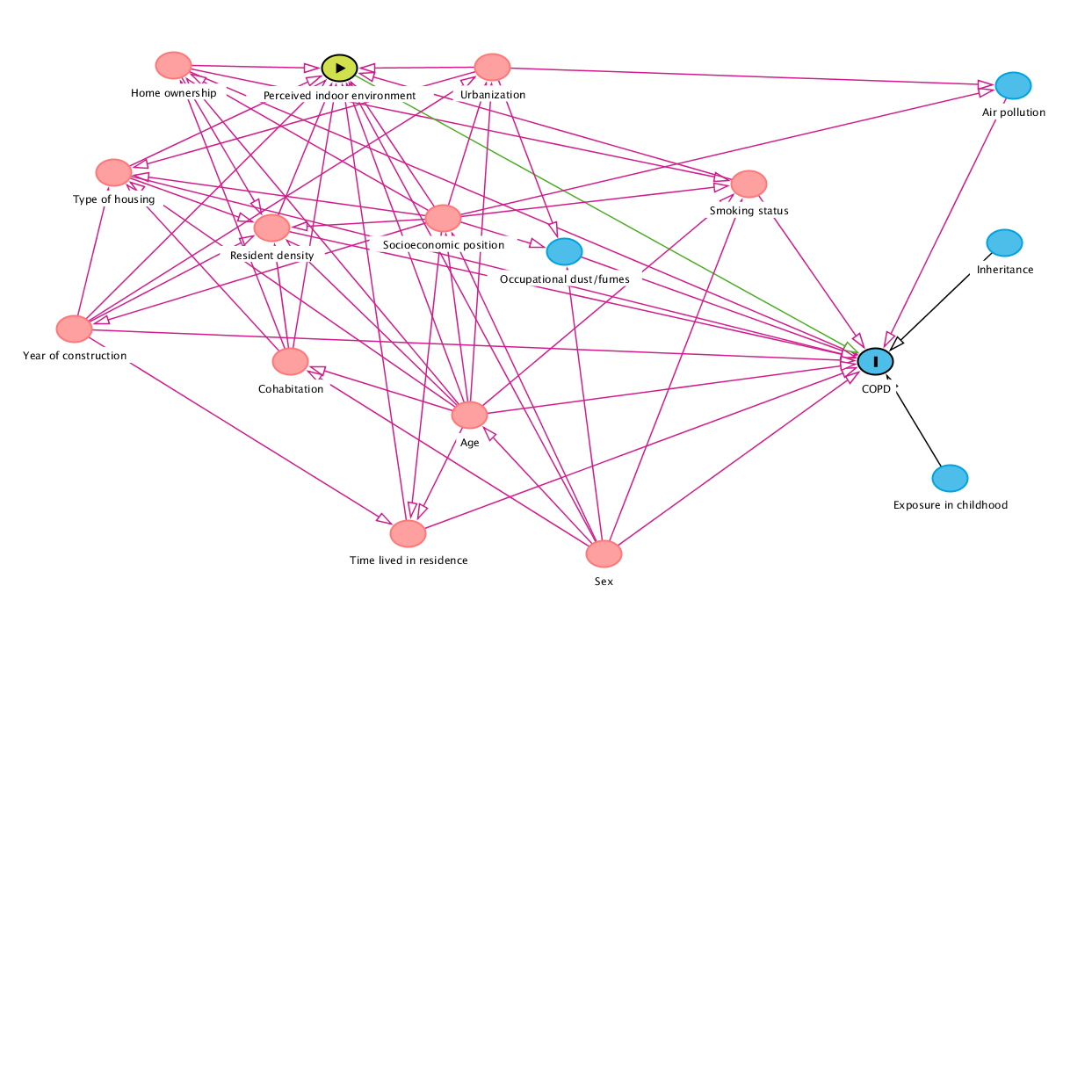**   |
| --- |
| **Figure S1f.** Directed Acyclic Graph (DAG) of the association between perceived indoor environment and Chronic Obstructive Pulmonary Disease (COPD). |
